# Quercetin attenuates acute inflammation and extracellular matrix remodeling after intracerebral hemorrhage: transcriptomics-informed network pharmacology and experimental validation

**DOI:** 10.3389/fphar.2026.1930434

**Published:** 2026-10-19

**Authors:** Jun Shu, Kexin Feng, Anqi Pan, Gang Li, Kaifeng Liu, Luqian Feng

**Affiliations:** 1 The People’s Hospital of the Qiandongnan Miao and Dong Autonomous Prefecture, Kaili, Guizhou, China; 2 Zunyi Medical University, Zunyi, Guizhou, China; 3 Guihang 300 Hospital Affiliated to Zunyi Medical University, Guiyang, Guizhou, China

**Keywords:** extracellular matrix remodeling, intracerebral hemorrhage, network pharmacology, neuroinflammation, Quercetin, transcriptomics

## Abstract

Intracerebral hemorrhage (ICH) induces secondary brain injury characterized by neuroinflammation, blood–brain barrier disruption, oxidative stress, and extracellular matrix (ECM) remodeling. Quercetin (QR), a natural flavonoid with anti-inflammatory and antioxidant activities, has shown neuroprotective potential; however, the molecular signatures associated with its effects in acute ICH remain incompletely defined. In this study, QR-related target prediction, ICH-associated gene mining, endoplasmic reticulum stress-related gene annotation, and 2 mouse brain transcriptomic datasets were integrated to prioritize QR-associated targets in ICH. Functional enrichment, protein–protein interaction, immune deconvolution, receiver operating characteristic (ROC), and molecular docking analyses were then performed. Key predictions were further evaluated in a collagenase-induced mouse ICH model treated with QR or dexamethasone for 24 h, and their disease relevance was assessed using paired perihematomal and remote brain tissues from ICH patients, with serum cytokine profiling performed in ICH patients and healthy controls. Twenty-nine candidate genes were identified and were mainly enriched in TNF and IL-17 signaling, leukocyte migration, stress-responsive programs, and ECM-associated processes. Seven consensus hub genes were prioritized, including *Il1b*, *Mmp9*, *Timp1*, *Myc*, *Fos*, *Hmox1*, and *Socs3*. Five hub genes in GSE113151 and six in GSE200575 showed high exploratory discriminative performance. Molecular docking suggested putative binding compatibility between QR and several hub proteins. *In vivo*, high-dose QR reduced brain water content after ICH. QR treatment suppressed perihematomal *Il1b*, *Mmp9*, and *Fos* expression, increased *Timp1* expression, and selectively upregulated *Hmox1* at the high dose, while reducing circulating IL-1β, IL-6, TNF-α, and MMP-9 levels. In human ICH samples, perihematomal tissues showed increased *IL1B*, *MMP9*, *TIMP1*, and *HMOX1* expression; Western blotting further showed increased IL-1β, MMP-9, and TIMP1 protein levels, and immunohistochemistry showed enhanced MMP-9 immunoreactivity. Circulating inflammatory cytokines were also elevated in patients with ICH. These findings suggest that QR is associated with attenuation of acute inflammatory and matrix-remodeling responses after experimental ICH, providing a pharmacological rationale for further target-focused mechanistic studies and long-term preclinical evaluation.

## 1 Introduction

Intracerebral hemorrhage (ICH) represents a devastating subtype of stroke with substantial mortality and long-term neurological disability (Greenberg et al., 2022; Sheth, 2022). After the primary mechanical injury caused by hematoma formation, secondary injury evolves through a complex cascade involving sterile inflammatory activation, redox imbalance, disruption of the neurovascular unit, perihematomal edema, and extracellular matrix (ECM) remodeling (Ohashi et al., 2023; Tao et al., 2024; Yan et al., 2023). Current guideline-based management mainly focuses on acute supportive care, blood pressure control, reversal of anticoagulation when appropriate, and selected surgical interventions; however, pharmacological therapies that effectively modify secondary injury remain limited (Greenberg et al., 2022). These unmet needs support the investigation of pharmacological strategies capable of modulating convergent inflammatory and tissue-remodeling pathways after ICH.

Quercetin (QR) is a widely distributed flavonoid found in fruits, vegetables, and medicinal plants. Its neuropharmacological effects have been linked to anti-inflammatory, antioxidant, anti-apoptotic, and barrier-protective activities across experimental models of neurological injury (Islam et al., 2021; Zhang et al., 2022; Zou et al., 2021). In a rodent model of ICH, QR was reported to improve post-hemorrhagic neurological outcomes, potentially through attenuation of inflammatory activation and apoptotic injury (Zhang et al., 2015). However, the molecular signatures associated with QR in acute ICH, and their relationship to disease-relevant inflammatory and ECM-remodeling targets, remain insufficiently characterized.

To address this gap, we combined transcriptomics-informed network pharmacology with experimental validation in a collagenase-induced mouse ICH model and disease-context assessment in human ICH samples. We focused on QR-associated inflammatory, cytoprotective, and matrix-remodeling targets and interpreted computational docking and immune-deconvolution outputs as hypothesis-generating evidence. Human perihematomal tissues and serum were used to evaluate the disease relevance of the prioritized targets rather than to infer QR-specific therapeutic efficacy in patients.

## 2 Materials and methods

### 2.1 Mapping QR targets in ICH via computational approaches

#### 2.1.1 Target identification

The chemical structure of QR was retrieved from PubChem for compound annotation. Putative QR targets were collected from SEA, SwissTargetPrediction, CTD, and OMIM, and merged after deduplication to yield 4,646 unique targets (Amberger and Hamosh, 2017; Daina et al., 2019; Kim et al., 2021; Keiser et al., 2007; Wiegers et al., 2025; Supplementary Table S1).

ICH-associated targets (n = 1,602) were compiled from GeneCards, MSigDB, CTD, and OMIM after removing duplicates (Stelzer et al., 2016; Liberzon et al., 2011; Supplementary Table S2). Because some databases annotate ICH under the broader term “cerebral hemorrhage,” all retrieved targets were interpreted in the context of ICH pathophysiology. Overlap analysis identified 657 common targets between QR- and ICH-associated gene sets, which were subsequently mapped to 642 murine orthologs using HomoloGene (v1.4.68; Supplementary Table S3). These orthologs were used for downstream network and enrichment analyses.

#### 2.1.2 ICH transcriptomic datasets and ERSRG reference set

Public mouse brain transcriptomic datasets GSE113151 and GSE200575 were retrieved from the Gene Expression Omnibus (GEO) using the GEOquery package in R (Barrett et al., 2013). All samples were derived from *Mus musculus* brain tissue. GSE113151 (GPL17021; 5 ICH and 5 control samples) was used as the discovery dataset, whereas GSE200575 (GPL24247; 5 ICH and 5 control samples) served as an independent validation cohort (Supplementary Table S4).

A reference list of endoplasmic reticulum stress–related genes (ERSRGs) was compiled from GeneCards, MSigDB, and published literature (Cheng et al., 2024; Zhang et al., 2024). After merging and deduplication, 2,558 ERSRGs were obtained and mapped to murine orthologs using HomoloGene, yielding 2,297 genes represented in the GSE113151 and GSE200575 datasets (Supplementary Table S5). This ERSRG list was used as a literature-derived reference annotation for candidate prioritization. Both datasets were processed and normalized using the limma package (v3.58.1; Ritchie et al., 2015).

#### 2.1.3 Differential expression analysis

Differential expression analysis was performed in GSE113151 using limma after stratifying samples into ICH and control groups. Genes meeting |log2FC| > 0.75 and nominal p < 0.05 were retained as exploratory differentially expressed genes (DEGs) for candidate screening and summarized in a volcano plot. Candidate key genes were then identified by overlapping exploratory DEGs, putative QR targets, and ERSRGs in a Venn diagram, and their expression patterns were visualized in a heatmap.

#### 2.1.4 Gene ontology (GO) and kyoto encyclopedia of genes and genomes (KEGG) enrichment analysis

To characterize the functional roles of key genes, GO and KEGG enrichment analyses were performed in R using clusterProfiler (v4.10.0; Yu et al., 2012). Terms were considered significant when BH-adjusted p < 0.05 and FDR q-value <0.25.

#### 2.1.5 Protein–protein interaction (PPI) network and hub gene identification

A PPI network was built in STRING (interaction score ≥0.4). Hub genes were ranked in Cytoscape (CytoHubba) using five centrality algorithms (MCC, Degree, MNC, EPC, and Closeness). The top 10 genes from each algorithm were extracted, and genes shared by all five top-10 lists were considered high-confidence hubs (Szklarczyk et al., 2019).

#### 2.1.6 Gene set enrichment analysis (GSEA) and gene set variation analysis (GSVA)

Genes in GSE113151 were ranked by log2FC (ICH vs. control) and analyzed by GSEA (clusterProfiler) using MSigDB C2 curated gene sets. Gene sets with BH-adjusted p < 0.05 and FDR q < 0.25 were considered significant. GSVA was performed using MSigDB C2: CP gene sets, and differential pathway activity was assessed with limma followed by BH correction (BH-adjusted p < 0.05).

#### 2.1.7 Receiver operating characteristic (ROC) and correlation analyses of hub genes

Hub-gene expression was examined in GSE113151 and externally validated in GSE200575. ROC curves and the area under the curve (AUC) were computed using pROC to evaluate the discriminative ability of each hub gene, with AUC values interpreted as low (0.5–0.7), moderate (0.7–0.9), or high (>0.9). In GSE113151, pairwise Spearman’s rank correlations among hub genes were calculated and visualized as a correlation heatmap using ggcorrplot. Correlation strength was interpreted as negligible (|r| < 0.3), weak (0.3–0.5), moderate (0.5–0.8), or strong (|r| > 0.8). Functional similarity (GO semantic similarity) among hub genes was assessed using GOSemSim.

#### 2.1.8 Computational estimation of immune-cell signatures and hub-gene associations

Immune-cell-related signatures in GSE113151 were computationally inferred from the expression matrix using CIBERSORT. Immune cell types with zero estimated proportions across samples were excluded. Composition estimates and group differences (ICH vs. control) were plotted using ggplot2. Spearman correlations were used to assess immune cell–immune cell relationships (pheatmap) and hub gene–immune cell associations (ggplot2).

#### 2.1.9 Molecular docking analysis of QR with hub proteins

To generate hypotheses regarding putative interactions between QR and hub proteins, hub-protein structures were predicted using AlphaFold 3 (v3.0; Abramson et al., 2024), and QR was subsequently docked to these structures using AutoDock Vina. The 3D structure of QR (CID: 5,280,343) was obtained from PubChem, and hub-protein sequences were retrieved from UniProt. The top-ranked AlphaFold model was used for downstream analysis. Vina-generated poses were visualized in PyMOL, and Vina scores were interpreted as follows: > −5 kcal/mol, weak binding; −7 to −5 kcal/mol, moderate binding; and < −7 kcal/mol, strong predicted binding.

### 2.2 Experimental validation in mice

#### 2.2.1 Mouse ICH model and experimental design

Male C57BL/6 mice (6–8 weeks old; 25–28 g) were purchased from Jiangsu Huachuang Xinnuo Co., Ltd. (SCXK(Su)2020–0009) and acclimated for 1 week under specific pathogen-free conditions at the Animal Experiment Center of Guizhou Medical University. All animal experiments were approved by the Animal Ethics Committee of Guizhou Medical University (approval No. 2402863) and are reported in accordance with the ARRIVE guidelines. All procedures were performed in accordance with the U.S. Public Health Service Policy on Humane Care and Use of Laboratory Animals and the NIH Guide for the Care and Use of Laboratory Animals.

For molecular and serum analyses, mice were randomly assigned (n = 6 per group) to six groups: sham-operated (Sham), ICH model (ICH), three ICH groups receiving QR at 25, 50, or 100 mg/kg per administration (ICH + QR25, ICH + QR50, and ICH + QR100), and an anti-inflammatory pharmacological comparator group treated with DEX at 2 mg/kg per administration (ICH + DEX).

Mice were anesthetized with 2% isoflurane via a precision vaporizer (Shanghai Puxin Instrument Technology Co., Ltd., China) and maintained on 1% isoflurane while secured in a stereotaxic frame (Beijing Easy-Jet Technology Co., Ltd., China). After shaving, the scalp was prepared with povidone–iodine, and a ∼2-cm midline incision was made to expose the skull and identify bregma. The striatum was targeted at stereotaxic coordinates 1.0 mm anterior and 2.0 mm lateral to bregma; a burr hole was drilled, and the needle was advanced to a depth of 4.0 mm. Collagenase VII (0.5 μL of a 0.5 U/μL solution; MCE, batch no. 776218, enzyme activity ≥1000 U/mg, USA) was injected to induce ICH, whereas the Sham group received an equivalent volume of sterile physiological saline (Guizhou Kolun Pharmaceutical Co., Ltd., batch no. E124072707, China). Each injection lasted approximately 5 min, followed by a 5-min needle dwell time before slow withdrawal. The burr hole was sealed with bone wax, and the incision was closed with sutures before the mice were returned to their home cages.

QR (MCE, batch no. 745316, purity 98.45%, USA) was prepared in 5% dimethyl sulfoxide (DMSO) and 95% sterile physiological saline and administered intraperitoneally twice, at 0.5 and 12 h after ICH induction. The low-, medium-, and high-dose groups received 25, 50, and 100 mg/kg QR per administration, respectively, corresponding to total administered doses of 50, 100, and 200 mg/kg over the 24-h experimental period. The first administration at 0.5 h was intended to provide early pharmacological intervention during the acute secondary-injury phase, whereas the second administration at 12 h provided repeat treatment within the 24-h observation period. The QR dose range was informed by a previous collagenase-induced rat ICH study in which QR at 5 and 50 mg/kg was evaluated, with more pronounced effects observed at 50 mg/kg (Zhang et al., 2015). Thus, 25, 50, and 100 mg/kg were selected as low, intermediate, and exploratory high doses, respectively, to evaluate dose-related responses in the present mouse model. Because the previous study differed from the present study in animal species and dosing protocol, the current regimen should be regarded as a literature-informed exploratory design rather than a direct replication.

DEX (Beijing Solarbio Technology Co., Ltd., batch no. ID0180, China) was prepared as a 10 mM stock solution in DMSO and diluted with sterile physiological saline to a final vehicle composition of 1% DMSO and 99% saline. DEX was administered intraperitoneally at 2 mg/kg per administration at 0.5 and 12 h after ICH induction, corresponding to a total administered dose of 4 mg/kg during the 24-h experimental period. DEX was used as an anti-inflammatory pharmacological comparator based on previous experimental evidence of its effects on hematoma-associated injury, brain edema, and inflammatory responses in collagenase-induced ICH models (Lema et al., 2004; Yang et al., 2011). Specifically, Lema et al. (2004) administered DEX at 1 mg/kg intraperitoneally at 1 and 24 h after surgery and reported improvements in neurological outcomes, hematoma volume, and inflammatory cellular responses. Yang et al. (2011) further reported that DEX reduced perihematomal edema and suppressed ICAM-1 and MMP-9 expression after experimental ICH. Thus, these previous studies provide experimental support for the use of DEX as an anti-inflammatory comparator but do not directly validate the present regimen of 2 mg/kg per administration at 0.5 and 12 h after ICH induction. The present DEX regimen should therefore be regarded as a literature-informed exploratory comparator design rather than a direct replication of previous protocols. Sham-operated and ICH model mice received an equivalent volume of sterile physiological saline intraperitoneally at the corresponding time points.

#### 2.2.2 Tissue collection and sample preparation

At 24 h after ICH induction, mice designated for molecular and serum analyses were deeply anesthetized with sodium pentobarbital (50 mg/kg, i.p.). After loss of the pedal withdrawal reflex, blood was collected by terminal cardiac puncture. Serum was isolated by centrifugation (3,000 × g, 10 min) for ELISA. The animals were then transcardially perfused with physiological saline to remove residual intravascular blood, and the brains were harvested and cut into approximately 1-mm-thick coronal sections. Under stereomicroscopic guidance, perihematomal tissue was collected from regions immediately adjacent to the visible hematoma margin, extending outward to a maximum radial distance of approximately 2–3 mm from that margin. The collected tissue typically comprised 2–3 coronal sections per animal, depending on the rostrocaudal extent of the hematoma. The 2–3 mm range refers to the maximal radial extent from the hematoma edge rather than a complete uniform ring of that width. The dissected tissue was immediately processed for total RNA extraction, and the wet weight or volume of each specimen was not separately recorded.

#### 2.2.3 Brain water content measurement

Brain water content was assessed by the wet–dry method in an independent cohort comprising the Sham, ICH, ICH + QR100, and ICH + DEX groups (n = 6 per group). At 24 h after ICH induction, the ipsilateral hemisphere was rapidly dissected, gently blotted, and weighed immediately to obtain the wet weight (WW). Samples were dried at 100 °C for 24 h until constant weight and then reweighed to obtain the dry weight (DW). Brain water content (%) was calculated as (WW − DW)/WW × 100.

#### 2.2.4 qRT-PCR

Tissues from the perihematomal rim in ICH mice and the corresponding region in sham mice were homogenized for total RNA extraction and reverse-transcribed into cDNA using a commercial RT SuperMix kit (Wuhan Servicebio, G3337, China). qRT-PCR was performed using a SYBR Green Master Mix (Wuhan Servicebio, G3326, China) on a real-time PCR system (Thermo Fisher Scientific, Applied Biosystems 7500 system, USA). Relative expression was quantified using the 2^−ΔΔCT^ method with β-actin as the reference gene (Supplementary Table S6).

#### 2.2.5 Serum cytokine quantification by ELISA

Serum cytokines were quantified by double-antibody sandwich ELISA using commercial kits (Shanghai Yiyun Biotechnology Co., Ltd., China): IL-1β (Catalog No. YZ-091201M), IL-6 (Catalog No. YZ-091260M), TNF-α (Catalog No. YZ-201406M), and MMP-9 (Catalog No. YZ-131316M), according to the manufacturer’s instructions.

### 2.3 Experimental validation in human subjects

#### 2.3.1 Human tissue collection and ethical approval

Human brain tissues were collected from patients with primary spontaneous ICH who underwent clinically indicated hematoma evacuation surgery at The People’s Hospital of the Qiandongnan Miao and Dong Autonomous Prefecture. Six ICH patients were enrolled, and all surgeries were performed within 24 h of symptom onset. During surgery, paired brain tissue specimens were obtained from each patient: (i) perihematomal tissue sampled from the rim immediately surrounding the hematoma (0–5 mm from the hematoma margin), and (ii) remote non-hemorrhagic parenchymal tissue collected along the surgical corridor at a distance of at least 2 cm from the hematoma margin, with no visible hemorrhage, contusion, or necrosis on gross inspection. All specimens were obtained from tissue that required removal during the clinically indicated surgical procedure; no additional brain tissue was resected solely for research purposes, nor was the surgical scope expanded for the study. The remote non-hemorrhagic tissue served as a within-patient control and does not represent normal brain tissue from healthy individuals. Each tissue specimen was divided for downstream analyses: one portion was snap-frozen and stored at −80 °C for qRT-PCR and Western blotting, and the remaining portion was fixed in 4% paraformaldehyde and paraffin-embedded for immunohistochemistry (IHC).

For peripheral measurements, venous blood samples were collected from six ICH patients (ICH group) within 24 h of symptom onset and from six healthy controls (HC group). HC were selected to approximate the patient group in age and sex and were screened to exclude active infection, immune-related diseases, and recent medication use that could affect inflammatory profiles. Serum was separated within 2 h of collection by centrifugation (1,500 × g, 10 min), aliquoted, and stored at −80 °C until cytokine measurement.

The study protocol involving human participants was approved by the Institutional Review Board/Ethics Committee of The People’s Hospital of the Qiandongnan (approval No. 2024026). Written informed consent was obtained from all participants or their legally authorized representatives. All procedures were conducted in accordance with the Declaration of Helsinki. Paired sampling (perihematomal vs. remote tissue from the same patient) was used to reduce inter-individual variability.

#### 2.3.2 qRT-PCR

Paired perihematomal and remote brain tissues were homogenized for total RNA extraction and reverse-transcribed into cDNA using a commercial RT SuperMix kit (Wuhan Servicebio, G3337, China). qRT-PCR was performed using a SYBR Green Master Mix (Wuhan Servicebio, G3326, China) on a real-time PCR system (Thermo Fisher Scientific, Applied Biosystems 7500 system, USA). Relative transcript abundance was expressed as 2^ΔCt^ using GAPDH as the reference gene (Supplementary Table S7).

#### 2.3.3 Western blotting

Snap-frozen perihematomal and remote brain tissues were lysed, and protein concentrations were determined using a BCA protein assay kit (Solarbio, Cat. No. PC0020, China). Equal amounts of protein were separated by SDS–PAGE and transferred onto PVDF membranes (Millipore, IPVH00010, Germany). After blocking, membranes were incubated with primary antibodies against IL-1β (Proteintech, Cat. No. 26048-1-AP, China), MMP-9 (Proteintech, Cat. No. 10375-2-AP, China), and TIMP1 (Qinke, Cat. No. AF7007, China), with β-actin (Bioss, Cat. No. bs-0061R, China) used as the loading control, followed by HRP-conjugated secondary antibody incubation (Beyotime, Cat. No. A0208, China). Immunoreactive bands were visualized using ECL reagents (Solarbio, Cat. No. P10100, China) and captured with a chemiluminescence imaging system.

#### 2.3.4 Immunohistochemistry (IHC)

Paraffin-embedded perihematomal and remote brain tissues were sectioned at 4 μm, deparaffinized, rehydrated, and subjected to antigen retrieval in sodium citrate buffer (Solarbio, C1032, China) under high-pressure heating for 13 min. Endogenous peroxidase activity was blocked with 3% H_2_O_2_, and sections were then incubated with a primary antibody against MMP-9 (Proteintech, 10375-2-AP, China) overnight at 4 °C, followed by an HRP-conjugated goat anti-rabbit secondary antibody (Sanying Biotechnology, RGAR011, China). Immunoreactivity was visualized using a DAB kit (Solarbio, DA1010, China), counterstained with hematoxylin, and mounted. Three non-overlapping fields per section were captured under identical imaging settings, and semi-quantitative analysis was performed using Image-Pro Plus 6.0.

#### 2.3.5 Serum cytokine quantification by flow cytometry (bead-based multiplex immunoassay)

Serum concentrations of IL-1β, IL-6, IL-10, IL-17A, and TNF-α were measured using commercial cytokine assay kits (Kuangbo Tongsheng Biotechnology Co., Ltd., Tianjin, China; lot No. 20251222) according to the manufacturer’s instructions. Briefly, serum samples and kit-provided standards were incubated with antibody-coated capture beads and detection reagents, and fluorescence data were acquired on a BD FACSCanto II flow cytometer (BD Biosciences, San Jose, CA, USA). Cytokine concentrations were calculated from standard curves generated in the same run.

### 2.4 Statistical methods

Statistical analyses were performed in R (v4.3.3) and GraphPad Prism (v10). Differential expression analysis used limma, with exploratory DEGs defined as |log2FC| > 0.75 and nominal p < 0.05. Enrichment analyses applied BH-adjusted p < 0.05 and FDR q < 0.25. ROC/AUC analyses used pROC; correlations were assessed by Spearman’s rank correlation. One-way ANOVA followed by Dunnett’s multiple-comparison test for comparisons with the ICH group, or Tukey’s test for all pairwise comparisons when appropriate. Paired human brain-tissue comparisons were performed using paired t-tests; for qRT-PCR data, statistical comparisons were performed on ΔCt values. Human serum cytokines were compared by Welch’s t-test on log-transformed values with Holm–Šídák correction. No animals were excluded except for predefined technical failures, and image quantification was performed blinded to group allocation where applicable. All tests were two-sided; p < 0.05 was considered statistically significant.

## 3 Results

### 3.1 Overall study design and analytical workflow

The study design and analytical workflow are shown in Figure 1.

**FIGURE 1:**
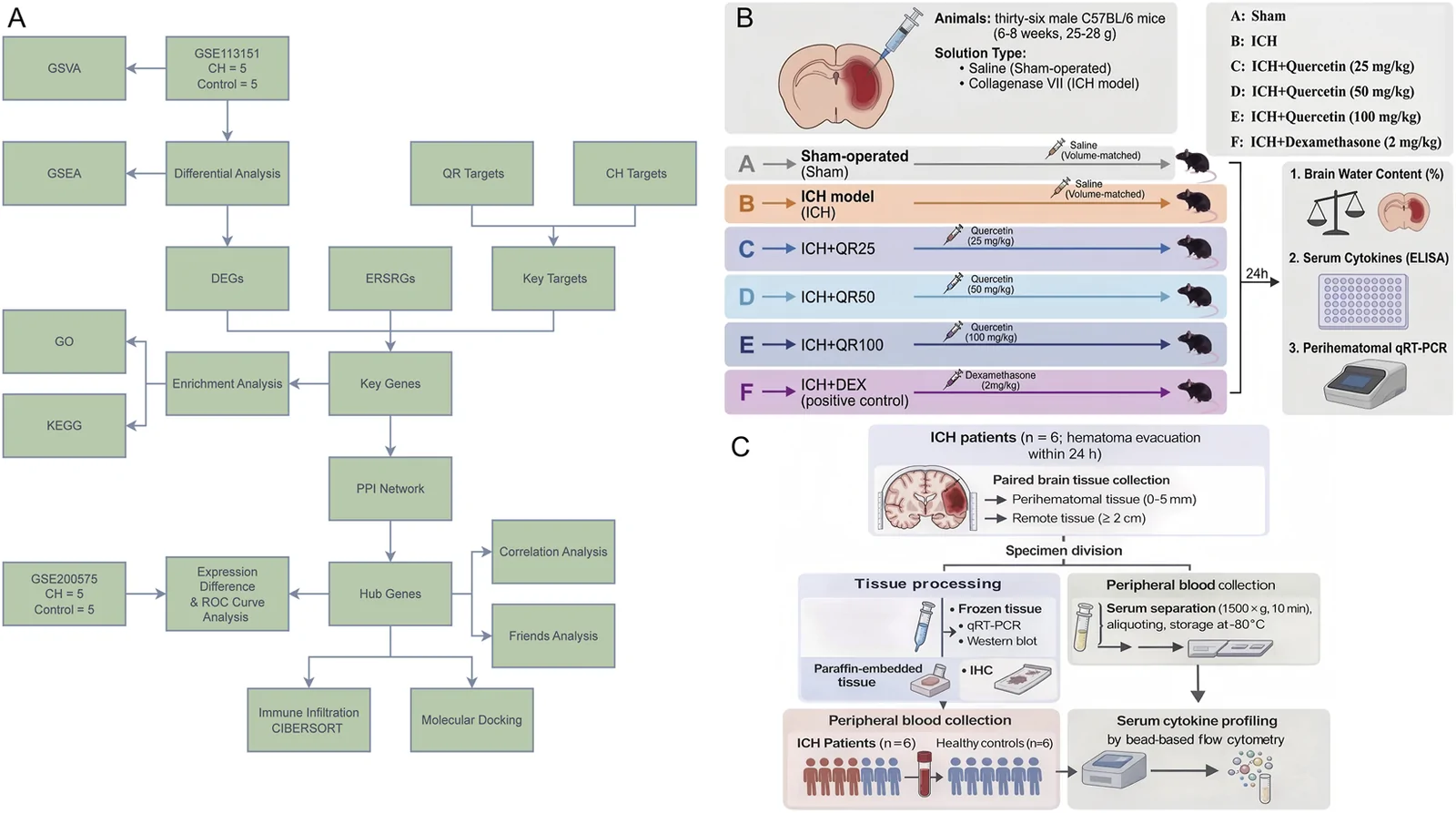
Study design and workflow. **(A)** Network pharmacology. **(B)** Mouse experiments. **(C)** Human disease-context assessment.

### 3.2 Target prediction and network construction

Cross-referencing 4,646 QR-related targets with 1,602 ICH-associated genes identified 657 overlapping genes (Figure 2A), which were mapped to 642 mouse orthologs for downstream analyses. A QR–target interaction network was constructed and visualized in Cytoscape (Figure 2B).

**FIGURE 2:**
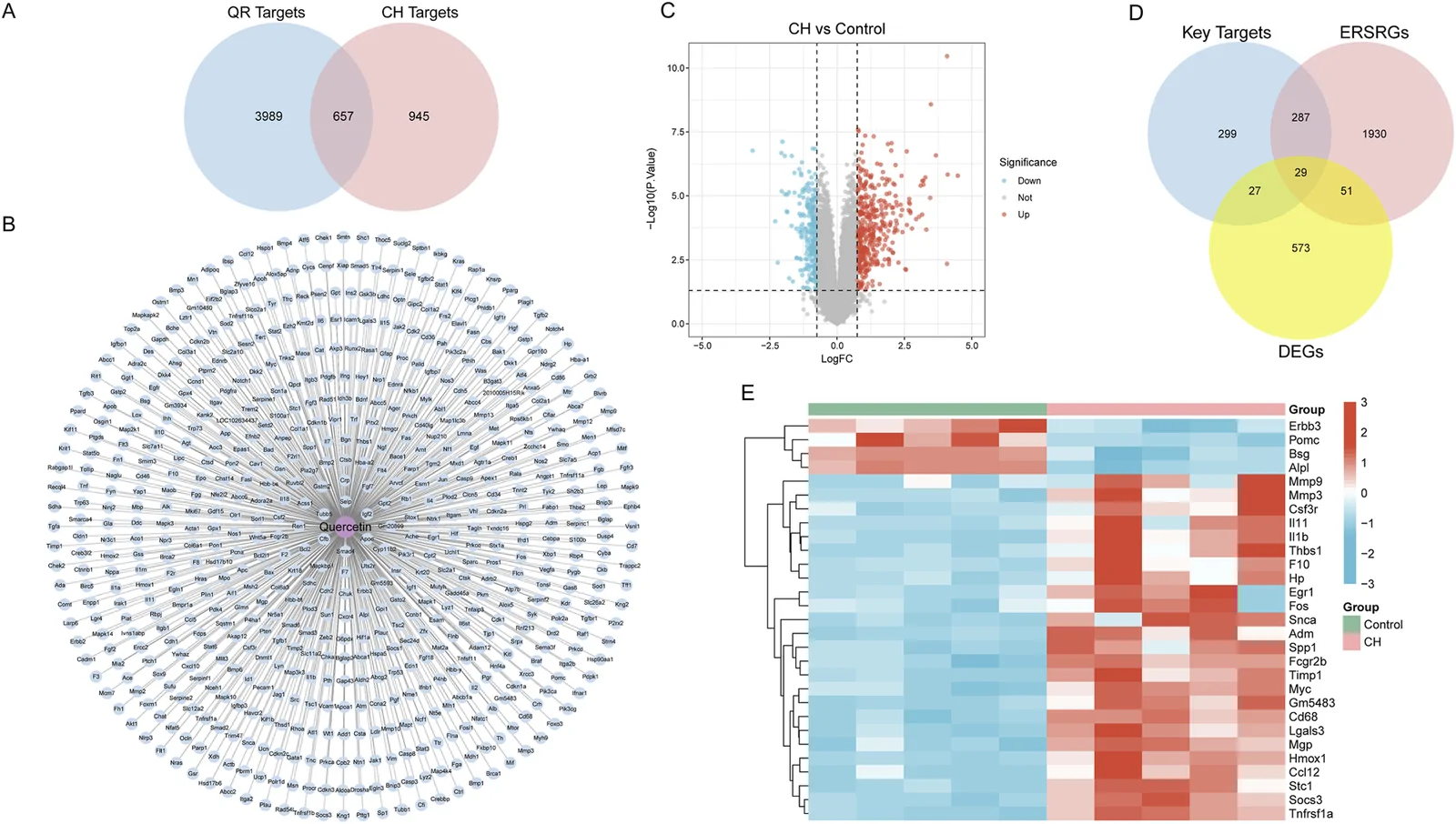
Identification of QR–ICH-associated targets and candidate key genes. **(A)** Venn diagram of QR-related targets and ICH-associated targets. **(B)** Compound-target network. **(C)** Volcano plot of DEGs between ICH (CH-labeled) and control samples in GSE113151. **(D)** Overlap among key targets, ERSRGs, and DEGs, yielding 29 genes. **(E)** Heatmap of the 29-gene expression patterns.

### 3.3 Differential expression analysis and identification of candidate key genes

In GSE113151, exploratory differential expression analysis identified 680 DEGs using |log2FC| > 0.75 and nominal p < 0.05, including 437 upregulated and 243 downregulated genes (Figure 2C). By overlapping these exploratory DEGs with QR targets and the ERSRG reference set, we identified 29 candidate key genes (Figure 2D; Supplementary Table S8), whose expression patterns differed between ICH and control samples (Figure 2E).

### 3.4 Functional landscape of candidate key genes in experimental ICH

GO and KEGG enrichment analyses of the candidate gene set (Supplementary Table S9) highlighted inflammatory cell trafficking and tissue remodeling. The top enriched GO terms included granulocyte chemotaxis, leukocyte chemotaxis, and myeloid leukocyte migration, together with positive regulation of angiogenesis. Additional enriched terms were related to collagen-containing extracellular matrix, secretory granules, cytokine activity, and extracellular matrix binding. KEGG analysis identified TNF and IL-17 signaling, along with vascular stress/atherosclerosis-related pathways (Figure 3A). Gene–term networks further summarized relationships across GO categories and KEGG (Figures 3B–E).

**FIGURE 3:**
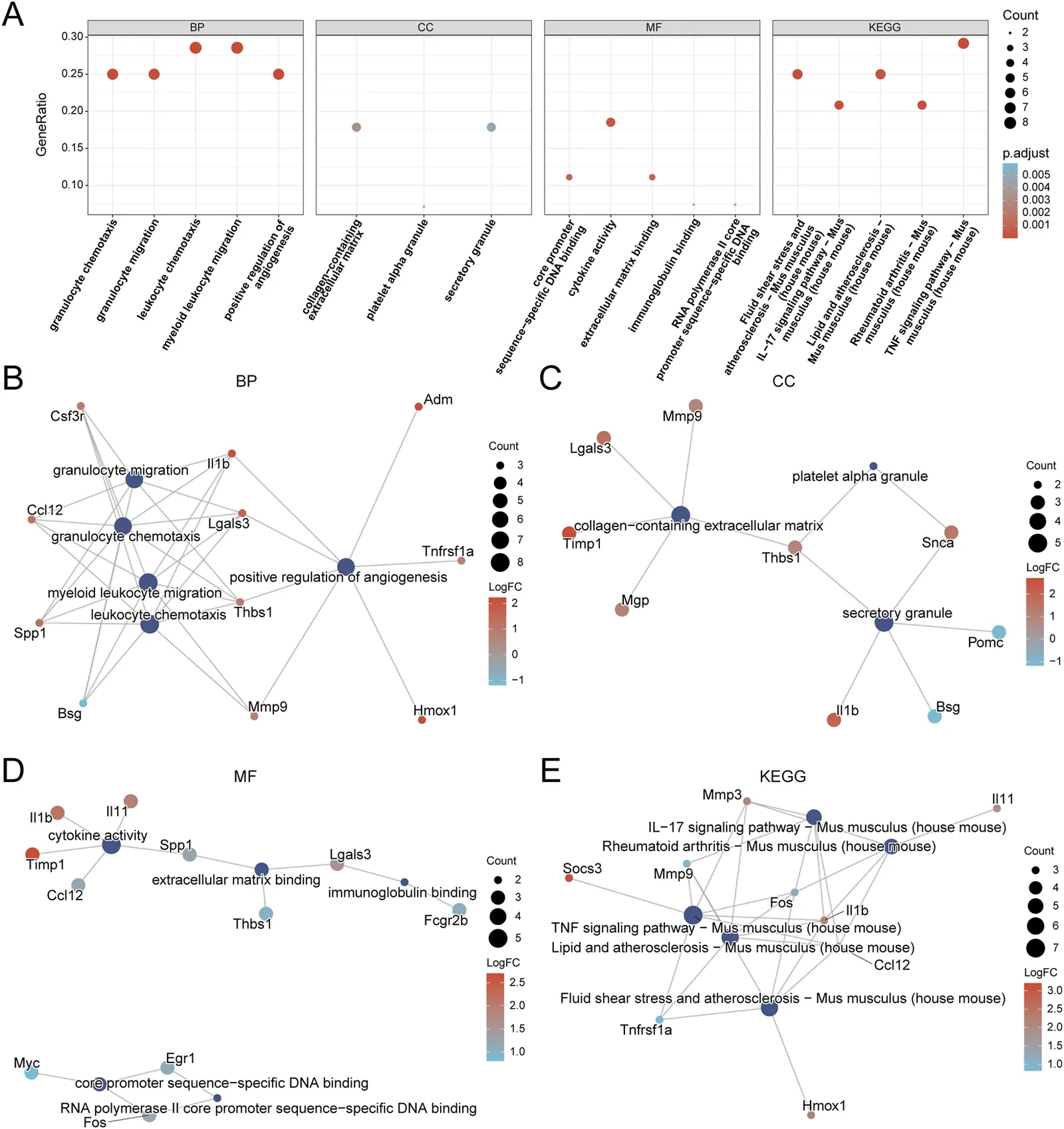
GO and KEGG enrichment analyses of the 29 key genes. **(A)** Bubble plots of enriched GO terms and KEGG pathways. Bubble size indicates gene count, and color indicates the BH-adjusted p value. **(B–E)** Gene–term networks for biological processes (BP; **(B)**) cellular components (CC; **(C)**) molecular functions (MF; **(D)**) and KEGG pathways **(E)** Term nodes are shown in dark blue, and gene nodes are colored by logFC (red, upregulated; blue, downregulated). Edges indicate gene–term links.

### 3.5 Consensus hub genes in the PPI network of key targets

Using STRING, we built a PPI network for the 29 candidate key genes (Figure 4A). Hub genes were ranked by CytoHubba using five centrality metrics, and the top 10 genes from each metric were shown (Figures 4B–F). The overlap among the five top-10 lists identified seven consensus hub genes—*Il1b, Mmp9, Timp1, Myc, Fos, Hmox1,* and *Socs3* (Figure 4G).

**FIGURE 4:**
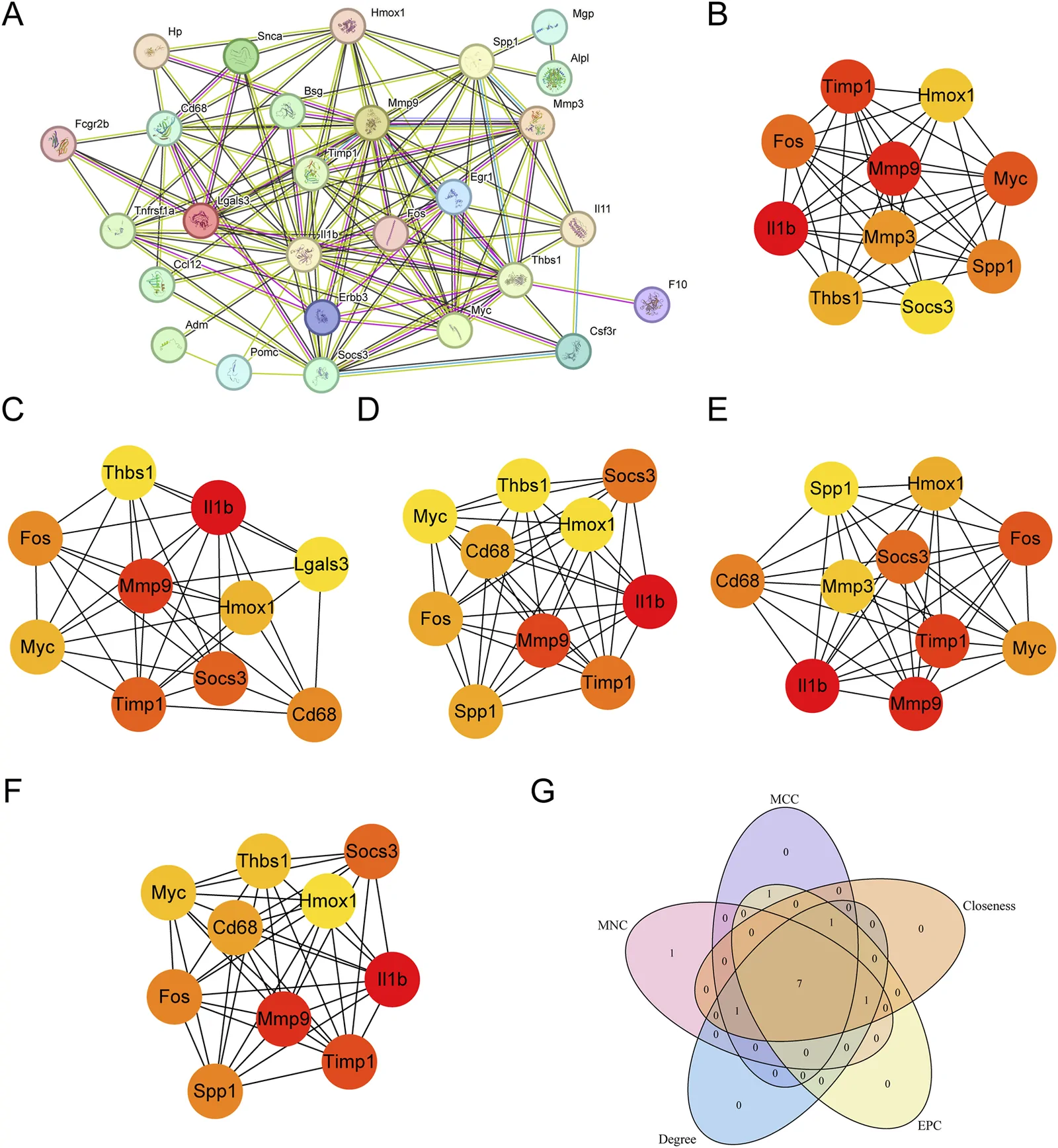
PPI network and hub-gene identification. **(A)** STRING PPI network of the 29 key genes. **(B–F)** Top 10 genes ranked by CytoHubba using MCC **(B)** MNC **(C)** Degree **(D)** EPC **(E)** and Closeness **(F)** node color transitions from red to yellow with decreasing scores. **(G)** Overlap of the top-10 lists, yielding seven consensus hub genes.

### 3.6 GSEA identifies coordinated pathway enrichment

GSEA revealed multiple gene sets significantly enriched in ICH versus controls (Figure 5A; Supplementary Table S10). Representative results (Figures 5B–E) highlighted immune/inflammatory and stress-responsive programs, indicating coordinated pathway enrichment.

**FIGURE 5:**
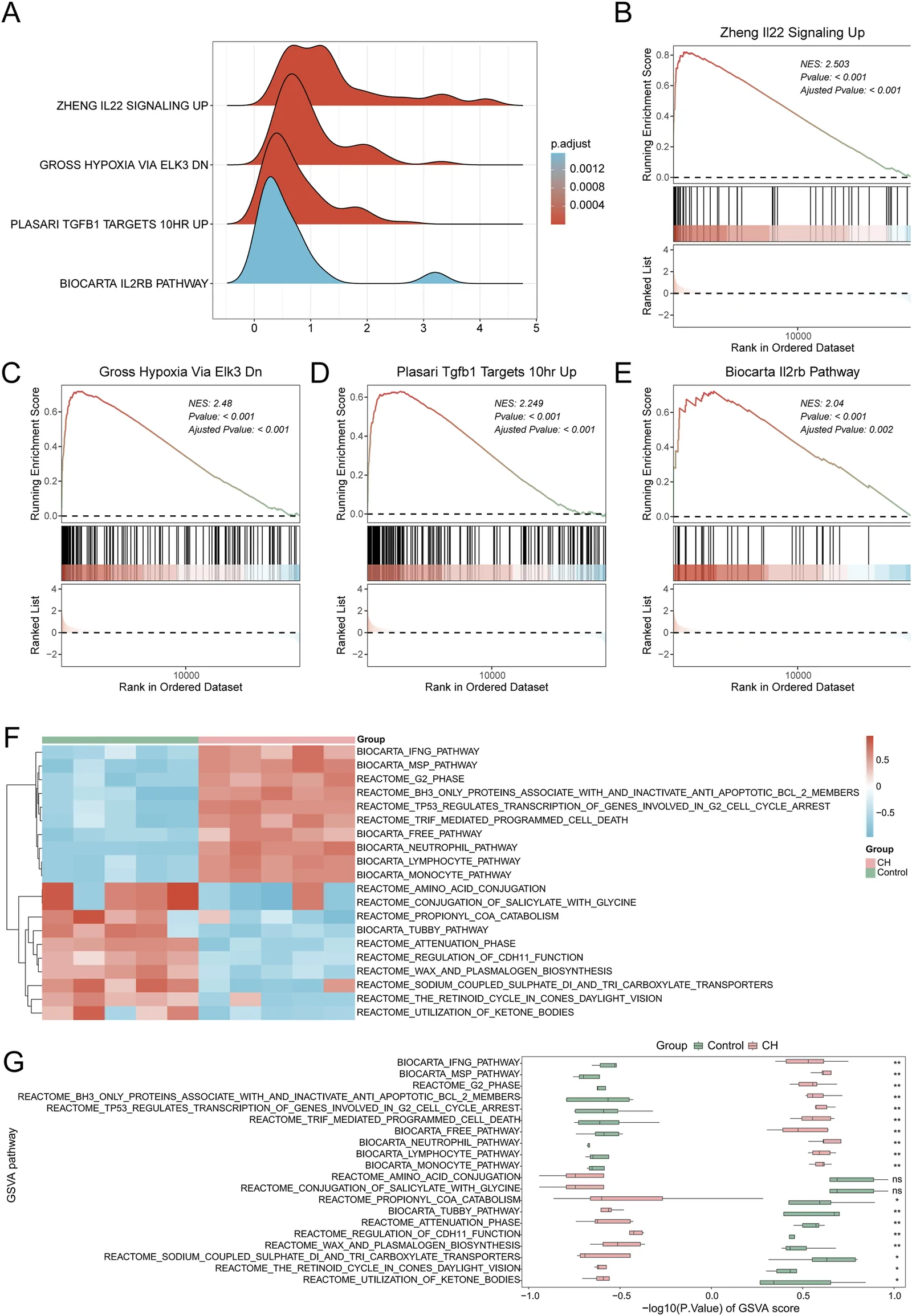
GSEA and GSVA pathway analyses in GSE113151. **(A)** Ridge plot summarizing significantly enriched MSigDB C2 gene sets (BH-adjusted p < 0.05 and FDR q < 0.25). **(B–E)** Enrichment plots for representative gene sets: Zheng IL22 Signaling_Up **(B)**, Gross Hypoxia Via Elk3 Dn **(C)**, Plasari Tgfb1 Targets 10h Up **(D)**, and BioCarta IL2rb Pathway **(E). (F)** Heatmap of the 10 most increased and 10 most decreased pathways (ranked by limma logFC; adjusted p < 0.05). **(G)**, Group comparison of representative pathways between ICH (CH-labeled) and control samples.

### 3.7 GSVA reveals sample-level pathway differences

GSVA showed broad pathway activity differences between ICH and control samples. The top ten increased and decreased pathways (ranked by limma logFC) are summarized (Figure 5F; Supplementary Table S11), with representative pathway scores shown in Figure 5G.

### 3.8 Hub gene expression and discriminative performance in GSE113151

In the discovery dataset GSE113151, among the seven hub genes, five hub genes (*Hmox1, Il1b, Myc, Socs3, Timp1*) showed significantly higher expression in ICH versus controls (all p < 0.01; Figure 6A), whereas *Fos* and *Mmp9* did not differ significantly between groups. ROC analysis based on the expression levels of the five significant genes indicated high discriminative performance between ICH and control samples, with AUCs >0.9 for each gene (Figures 6B–D).

**FIGURE 6:**
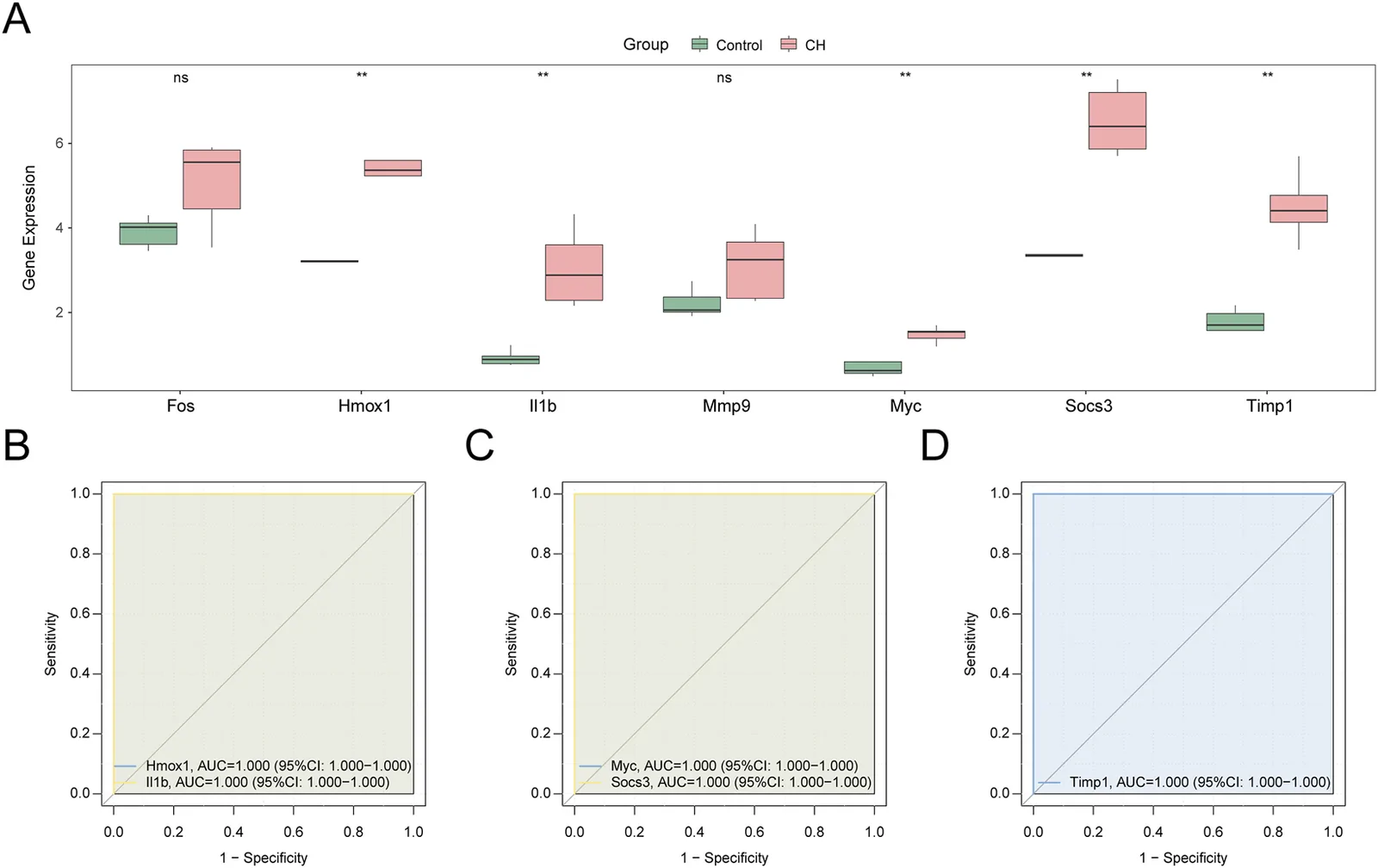
Hub gene expression and ROC analysis in GSE113151. **(A)** Expression differences of the seven hub genes between ICH (CH-labeled) and control samples. **(B–D)** ROC curves for hub genes with AUC >0.9. *p < 0.05, **p < 0.01.

### 3.9 External validation in GSE200575

In the independent GSE200575 cohort, six of the seven consensus hub genes (*Hmox1*, *Il1b*, *Mmp9*, *Myc*, *Socs3*, and *Timp1*) were differentially expressed between ICH and controls (*Hmox1*, *Mmp9*, *Myc*, *Socs3*, and *Timp1*: p < 0.01; *Il1b*: p < 0.05; Figure 7A). ROC analyses showed that these six genes achieved high discriminative performance (all AUC >0.9; Figures 7B–D).

**FIGURE 7:**
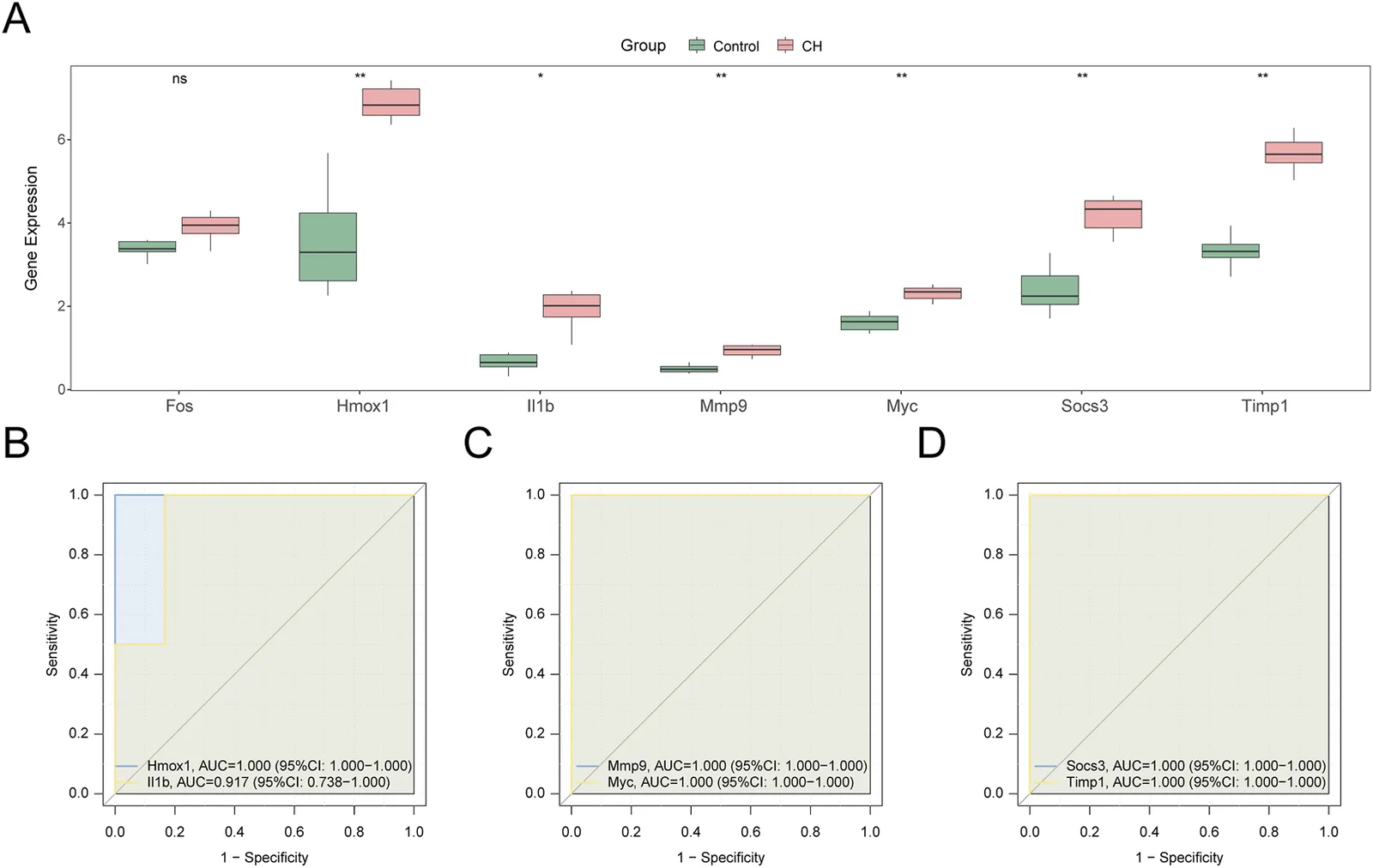
Hub gene expression and ROC analysis in GSE200575. **(A)** Expression differences of the seven hub genes between ICH (CH-labeled) and control samples. **(B–D)** ROC curves for hub genes with AUC >0.9. *p < 0.05, **p < 0.01.

### 3.10 Correlation and functional similarity analyses

In GSE113151, expression levels of the seven hub genes were positively correlated by Spearman’s rank correlation analysis (Figure 8A), with the strongest association observed between *Timp1* and *Myc* (r = 0.94, p < 0.001). Functional similarity analysis further showed that *Fos* had the highest similarity scores within the hub-gene set (Figure 8B).

**FIGURE 8:**
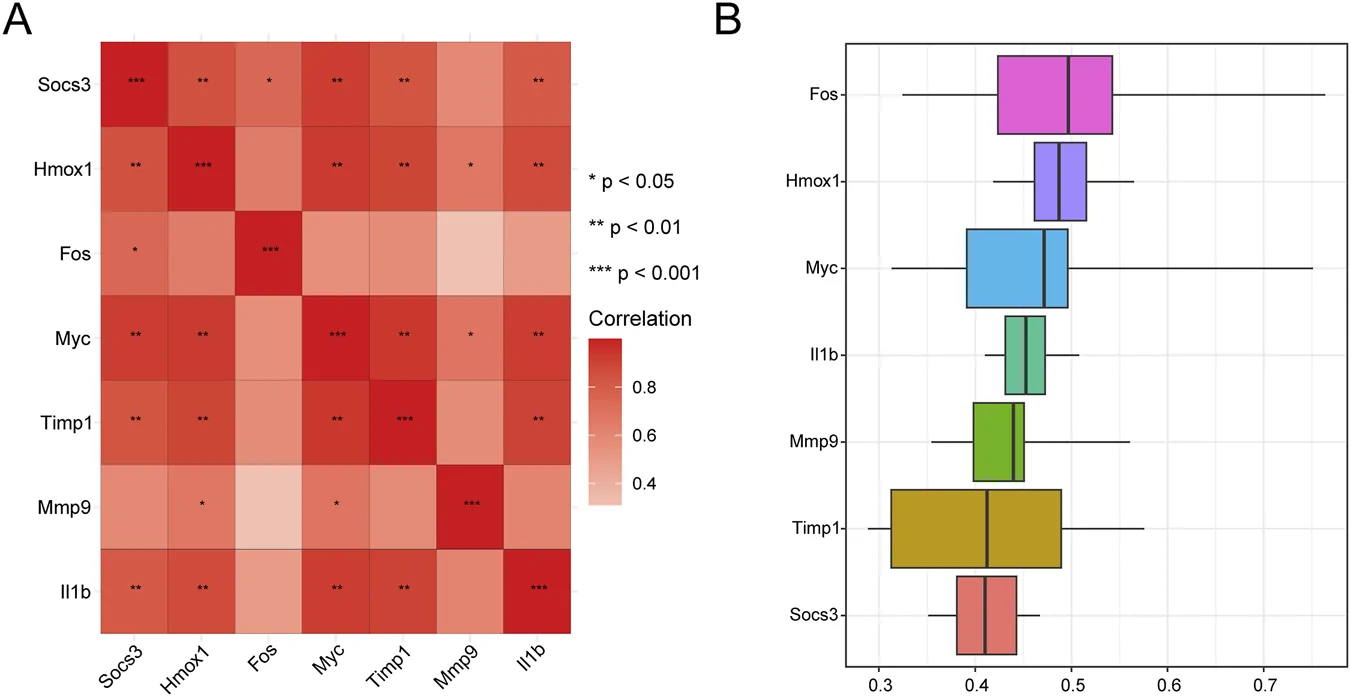
Hub-gene co-expression and functional similarity in GSE113151. **(A)** Spearman correlation heatmap. **(B)** GO semantic similarity scores among hub genes shown as boxplots. *p < 0.05, **p < 0.01, ***p < 0.001.

### 3.11 Estimated immune-cell signatures and hub-gene correlations

Immune-cell fractions (22 immune cell types) were estimated in GSE113151 (Figure 9A). Compared with controls, ICH samples showed higher fractions of M1 macrophages and memory CD8 T cells (both p < 0.01), with additional differences in eosinophils and resting NK cells (p < 0.05; Figure 9B). Correlation analysis revealed that eosinophils were most strongly positively correlated with memory CD8 T cells (r = 0.80) and most strongly negatively correlated with M1 macrophages (r = −0.91) (Figure 9C). Among hub genes, *Socs3* showed the strongest associations with immune-cell fractions, correlating positively with resting NK cells (r = 0.87) and negatively with eosinophils (r = −0.95) (both p < 0.001; Figure 9D).

**FIGURE 9:**
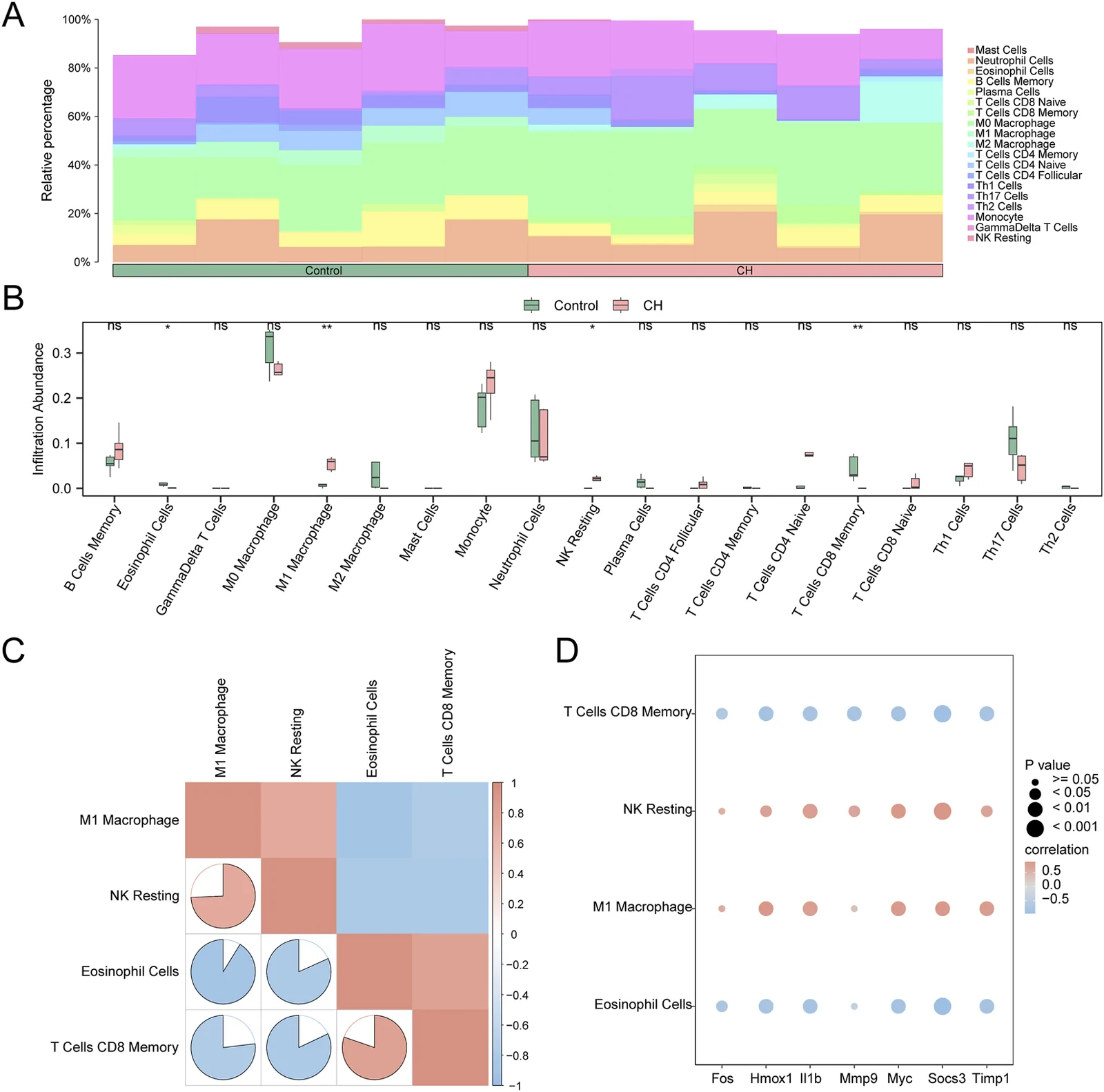
Immune cell infiltration analysis in GSE113151. **(A)** Stacked bar plot showing the relative proportions of immune cell types across samples. **(B)** Group comparison of immune-cell infiltration between ICH (CH-labeled; pink) and control samples (green). **(C)** Spearman correlation heatmap of immune-cell fractions. **(D)** Bubble plot showing Spearman correlations between hub-gene expression and immune-cell infiltration; color indicates correlation direction and magnitude (red, positive; blue, negative). *p < 0.05, **p < 0.01, ***p < 0.001.

### 3.12 Predicted docking of QR with hub proteins

Docking analyses suggested putative binding compatibility between QR and the seven hub proteins (Figures 10A–G; Supplementary Table S12). Strong predicted binding was observed for *Fos* (−7.85 kcal/mol), *Hmox1* (−7.81), *Il1b* (−7.57), *Mmp9* (−7.46), and *Timp1* (−7.27), whereas *Socs3* (−6.69) and *Myc* (−5.43) showed moderate predicted affinity. These *in silico* results suggest putative QR–hub protein interactions, while direct target engagement requires orthogonal biochemical validation.

**FIGURE 10:**
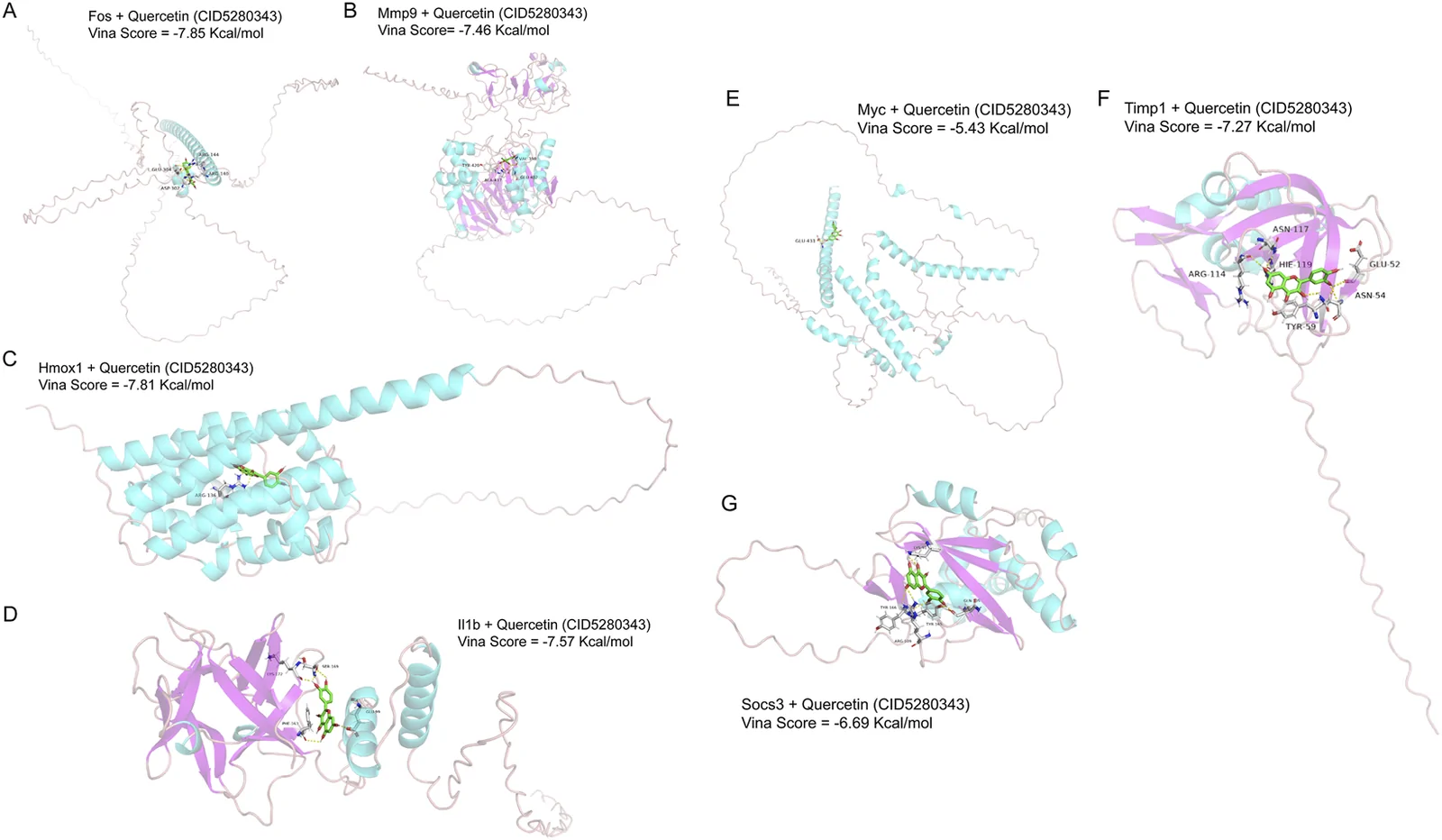
Molecular docking of QR with hub proteins. **(A–G)** Predicted binding poses of QR with Fos **(A)** Mmp9 **(B)** Hmox1 **(C)** Il1b **(D)** Myc **(E)** Timp1 **(F)** and Socs3 **(G)** Yellow dashed lines indicate hydrogen bonds. Docking scores were interpreted as described in Methods.

### 3.13 QR reduces brain edema assessed by wet–dry weight

At 24 h after ICH induction, brain water content was significantly increased in the ICH group compared with sham-operated controls (p < 0.0001). QR (100 mg/kg) significantly reduced brain water content (p < 0.001 vs. ICH), and DEX also reduced brain water content (p < 0.0001 vs. ICH), with no significant difference between the QR100 and DEX groups (Figure 11A).

**FIGURE 11:**
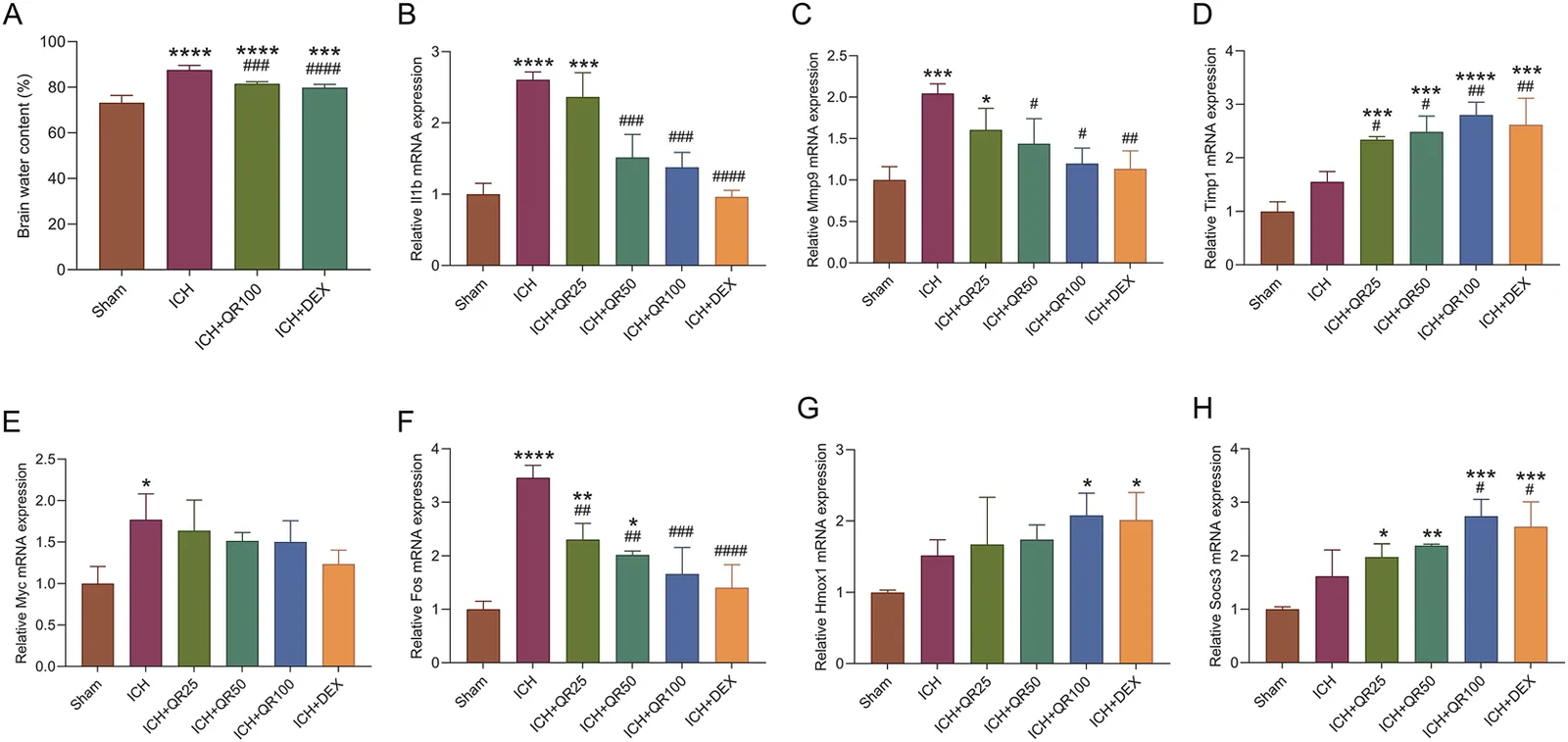
Dose-dependent effects of QR on hub-gene expression after ICH. **(A)** Brain water content (n = 6/group). **(B–H)** Perihematomal mRNA expression of *Il1b, Mmp9, Timp1, Myc, Fos, Hmox1, and Socs3* (n = 6/group). *p < 0.05, **p < 0.01, ***p < 0.001, ****p < 0.0001 vs. Sham; #p < 0.05, ##p < 0.01, ###p < 0.001, ####p < 0.0001 vs. ICH.

### 3.14 Dose-dependent modulation of hub-gene mRNA by QR

Perihematomal expression of the seven hub genes was quantified by qRT-PCR at 24 h after ICH induction. Compared with sham-operated controls, *Il1b* and *Mmp9* mRNA were strongly upregulated in the ICH group (Figures 11B,C; p < 0.0001 and p < 0.001, respectively). Medium- and high-dose QR, as well as DEX, attenuated *Il1b* induction (Figure 11B; p < 0.001 vs. ICH), whereas low-dose QR had no significant effect on *Il1b*. All three QR doses and DEX reduced *Mmp9* expression (Figure 11C; p < 0.05–0.01 vs. ICH). *Timp1* mRNA was not significantly elevated in the ICH group relative to sham-operated controls, but was significantly increased by all QR doses and DEX (vs. sham: low- and medium-dose QR and DEX, p < 0.001; high-dose QR, p < 0.0001), with all treatment groups showing significantly higher expression than the ICH group (low- and medium-dose QR: p < 0.05; high-dose QR and DEX: p < 0.01; Figure 11D). *Myc* mRNA increased slightly after ICH (p < 0.05 vs. sham) but was not significantly altered by any QR dose or DEX (Figure 11E). *Fos* mRNA was markedly induced by ICH (p < 0.0001 vs. sham); all QR doses and DEX reduced *Fos* expression (all p < 0.01–0.0001 vs. ICH; Figure 11F), with the low- and medium-dose QR groups remaining significantly above sham levels (p < 0.05–0.01), while high-dose QR and DEX restored expression to near-sham levels. *Hmox1* mRNA increased selectively with high-dose QR and DEX (Figure 11G; p < 0.05 vs. sham). *Socs3* mRNA showed only a mild, non-significant rise after ICH, but increased in a dose-dependent manner with QR treatment (QR25: p < 0.05; QR50: p < 0.01; QR100 and DEX: p < 0.001 vs. sham; Figure 11H); high-dose QR and DEX also exceeded ICH levels (p < 0.05 vs. ICH).

### 3.15 QR dose-dependently modulates systemic inflammatory markers following ICH

Serum levels of IL-1β, IL-6, MMP-9, and TNF-α were quantified by ELISA at 24 h after ICH induction (Figures 12A–D). Compared with the sham group, the ICH group showed marked systemic inflammation, with significant increases in IL-1β (p < 0.0001), IL-6 (p < 0.001), TNF-α (p < 0.001), and MMP-9 (p < 0.001). QR exerted a dose-dependent anti-inflammatory effect. At 25 mg/kg, all four markers remained significantly elevated versus sham and did not differ significantly from the ICH group. At 50 mg/kg, QR significantly reduced IL-1β (p < 0.01), IL-6 (p < 0.05), and TNF-α (p < 0.05) versus ICH, while these cytokines remained significantly higher than sham (p < 0.05); MMP-9 was also reduced versus ICH (p < 0.05) and was not statistically different from sham. At 100 mg/kg, QR produced the strongest effect, lowering IL-1β (p < 0.001) and reducing IL-6, TNF-α, and MMP-9 (all p < 0.01) versus ICH, with no significant differences versus sham for any marker. DEX showed a similar pattern.

**FIGURE 12:**
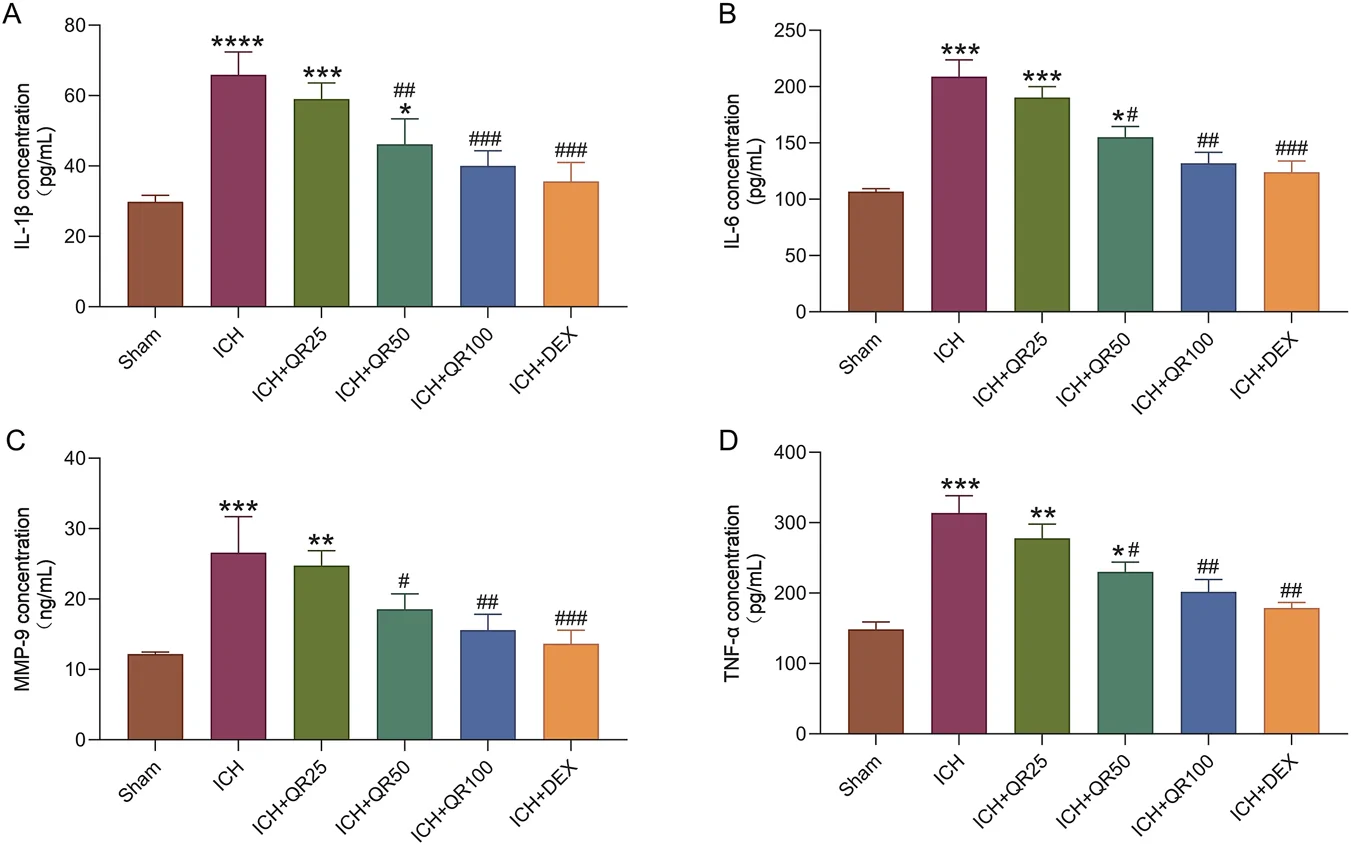
Dose-dependent effects of QR on systemic inflammatory markers after ICH. **(A–D)** Serum IL-1β, IL-6, MMP-9, and TNF-α (n = 6/group).*p < 0.05, **p < 0.01, ***p < 0.001, ****p < 0.0001 vs. Sham; #p < 0.05, ##p < 0.01, ###p < 0.001, ####p < 0.0001 vs. ICH.

### 3.16 Disease-relevant upregulation of inflammatory and injury-related genes in human perihematomal brain tissues

Paired perihematomal and remote brain tissues from ICH patients (n = 6 pairs) were analyzed by qRT-PCR (Figures 13A–D). Compared with matched remote tissues, perihematomal tissues showed significantly increased mRNA expression of *IL1B*, *MMP9*, *TIMP1*, and *HMOX1*. *MMP9* showed the most pronounced increase (p < 0.001), whereas *IL1B* and *HMOX1* were also significantly elevated (both p < 0.01). *TIMP1* showed a more moderate but still significant increase (p < 0.05). These findings indicate activation of inflammatory, matrix-remodeling, and cytoprotective transcriptional programs in human perihematomal tissue.

**FIGURE 13:**
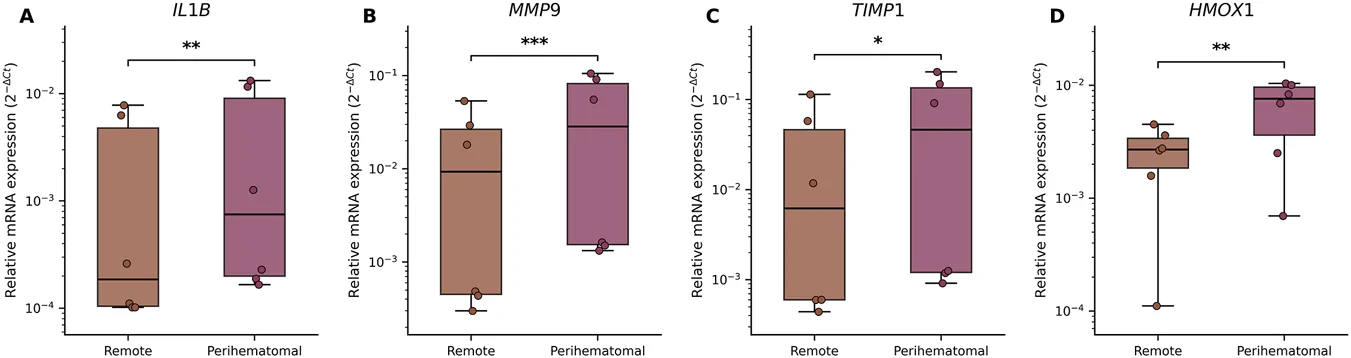
Inflammatory and injury-related gene expression in human ICH brain tissues. Relative mRNA expression of **(A)** *IL1B*, **(B)** *MMP9*, **(C)** *TIMP1*, and **(D)** *HMOX1* in remote and perihematomal brain tissues from six patients with ICH (n = 6 per tissue region). Data are presented as box-and-whisker plots with individual observations shown as points. *p < 0.05, **p < 0.01, ***p < 0.001.

### 3.17 Increased expression of inflammatory and matrix-remodeling proteins in human perihematomal brain tissues

To further validate the transcriptional findings at the protein level, Western blotting was performed in paired perihematomal and remote brain tissues from the same patients (Figures 14A–D). Representative blots showed increased protein expression of IL-1β, MMP-9, and TIMP1 in perihematomal tissues relative to the matched remote tissues. Among these proteins, MMP-9 showed the most pronounced increase, whereas IL-1β and TIMP1 were also elevated in perihematomal tissues. Densitometric analysis based on all paired samples confirmed significant increases in IL-1β (p < 0.01), MMP-9 (p < 0.001), and TIMP1 (p < 0.001). These protein-level changes were consistent with the qRT-PCR findings and further supported enhanced inflammatory activation and matrix remodeling in human ICH brain tissues.

**FIGURE 14:**
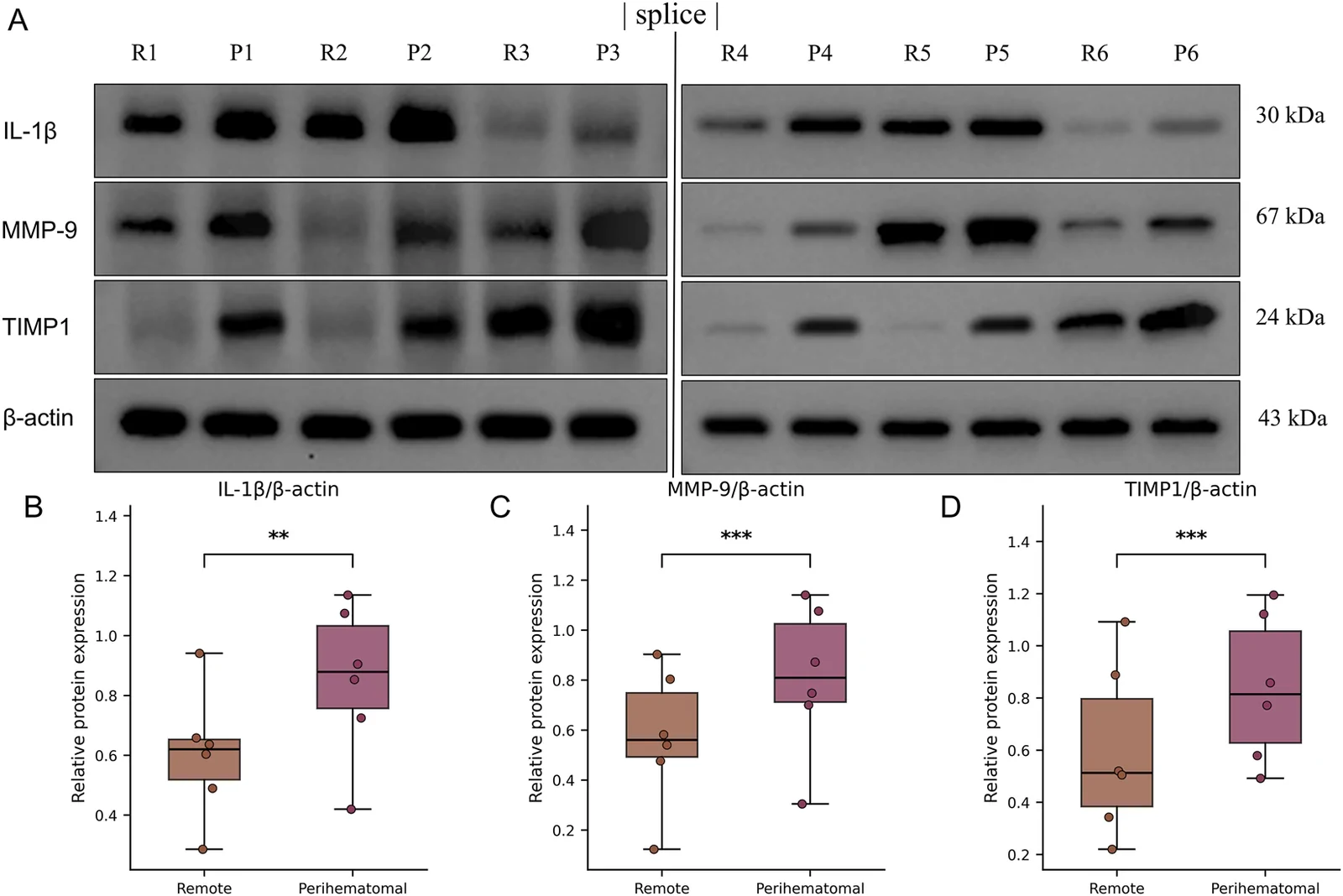
Western blot validation in human ICH brain tissues. **(A)** Representative western blots of IL-1β, MMP-9, TIMP1, and β-actin in remote (R) and perihematomal (P) brain tissues from six patients with ICH (n = 6 per tissue region). Where non-adjacent lanes were assembled for presentation, vertical lines indicate splice boundaries, and the corresponding uncropped blots are provided in the Supplementary Material. **(B–D)** Quantification of IL-1β/β-actin, MMP-9/β-actin, and TIMP1/β-actin, respectively. Data are presented as box-and-whisker plots with individual observations shown as points. **p < 0.01, ***p < 0.001.

### 3.18 Increased MMP-9 immunoreactivity in human perihematomal brain tissues

Immunohistochemical (IHC) staining provided additional spatial evidence for MMP-9 upregulation in human perihematomal brain tissues (Figure 15A). Compared with matched remote tissues, perihematomal tissues showed markedly stronger MMP-9 immunoreactivity, with prominent positive staining in perihematomal cells. Semi-quantitative image analysis confirmed a significant increase in the MMP-9-positive area (p < 0.001, Figure 15B). These findings were spatially consistent with the qRT-PCR and WB results.

**FIGURE 15:**
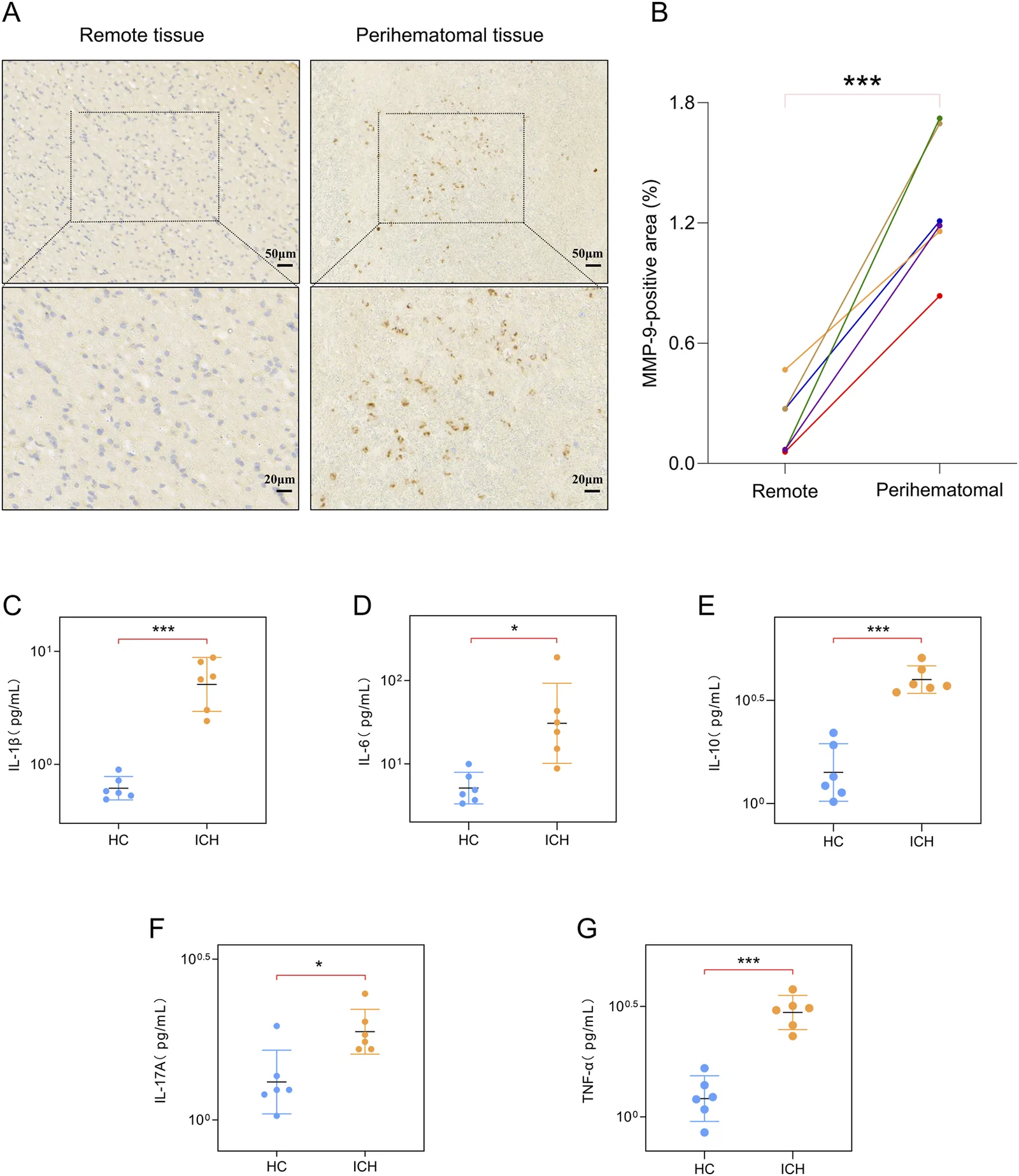
MMP-9 immunoreactivity in paired human ICH tissues and serum cytokine profiles in ICH patients. **(A)** Representative IHC images of MMP-9 in matched remote and perihematomal brain tissues. **(B)** Semi-quantitative analysis of MMP-9-positive area in paired remote and perihematomal tissues (n = 6 pairs). Each dot represents one patient, and lines connect matched pairs. **(C–G)** Serum concentrations of IL-1β **(C)**, IL-6 **(D)**, IL-10 **(E)**, IL-17A **(F)**, and TNF-α **(G)**, in ICH patients and healthy controls (n = 6 per group). Dots represent individual subjects; lines indicate geometric mean ±95% CI; y-axes are shown on a log10 scale. Serum cytokines were analyzed using Welch’s t-test on log-transformed data with Holm–Šídák adjustment across five cytokines. *p_adj <0.05, **p_adj <0.01, ***p_adj <0.001.

### 3.19 Inflammatory cytokine profiles in ICH patients

Serum concentrations of all five cytokines were significantly elevated in ICH patients compared with healthy controls (HC; n = 6 per group), based on Holm–Šídák-adjusted p values. IL-1β, IL-10, and TNF-α showed highly significant increases (all adjusted p < 0.001), while IL-6 and IL-17A were also significantly elevated (both adjusted p = 0.014) (Figures 15C–G; Supplementary Table S17).

## 4 Discussion

In ICH, the hematoma causes the initial mass effect, whereas secondary injury cascades drive ongoing neurological deterioration and critically influence clinical outcomes (Greenberg et al., 2022). Despite advances in supportive care, effective disease-modifying therapies remain limited. QR is a plant-derived flavonoid with well-established anti-inflammatory and antioxidant activities and has shown neuroprotective potential in experimental studies (Cai et al., 2025; Zhang et al., 2015; Zou et al., 2021), yet its key targets and regulatory mechanisms in ICH remain incompletely understood. In the present study, we integrated network pharmacology with omics-guided prioritization and experimental validation to identify QR-associated targets and pathways relevant to acute ICH. Overall, our findings suggest that QR treatment is associated with attenuation of acute inflammatory and ECM-remodeling responses after experimental ICH, supporting further target-focused and long-term preclinical investigation.

Using a transcriptomics-informed network pharmacology approach, we prioritized a compact set of QR-linked regulators in ICH by intersecting predicted QR targets with ICH-associated DEGs, highlighting hub nodes involved in inflammatory signaling (*Il1b*, *Mmp9*), ECM regulation (*Timp1*), heme/oxidative-stress responses (*Hmox1*), and stress-responsive transcription (*Fos*, *Myc*, *Socs3*). Together, these hubs defined a target-associated network involving inflammatory signaling, ECM regulation, and stress-response pathways. IL-1β and MMP-9 are closely linked to inflammatory amplification and BBB disruption in ICH (Guan et al., 2025), whereas TIMP-1 may limit excessive proteolysis and vascular leakage (Ahmadighadykolaei et al., 2023), and Hmox1 may contribute to heme catabolism and antioxidant defense. Immune deconvolution further suggested an ICH-associated shift toward pro-inflammatory cell states, in line with the prominent immune-cell responses described after ICH (Taskin and Yao, 2025), and identified notable hub–immune associations, particularly for *Socs3*. Consistent with this framework, QR-associated transcriptional changes in perihematomal tissue were accompanied by attenuation of circulating inflammatory mediators *in vivo*, supporting a coordinated anti-inflammatory response across central and peripheral compartments after ICH. In silico docking further generated hypotheses regarding possible QR–hub protein compatibility, in line with the polypharmacological properties of flavonoids (Pinzi and Rastelli, 2019), although these predictions should not be interpreted as evidence of direct biochemical binding.

A previous network pharmacology study based primarily on human databases identified TP53, STAT3, AKT1, SRC, JUN, TNF, and IL6 as core candidate targets of QR in ICH (Zhang et al., 2024), which differs from the seven hub genes prioritized in the present study (*Fos, Hmox1, Il1b, Mmp9, Myc, Socs3,* and *Timp1*). This discrepancy likely reflects differences in species background, database coverage, target-selection criteria, disease-specific transcriptomic constraints, and bioinformatic workflows. The previous study identified overlapping QR- and ICH-associated targets from human-oriented pharmacological and disease databases and prioritized core nodes using Degree, Closeness, and Betweenness centrality measures. In contrast, our analysis mapped QR–ICH-associated genes to murine orthologs and further constrained candidate selection by differential expression in the mouse ICH brain dataset GSE113151, an endoplasmic reticulum stress-related gene reference set, and the consensus of five CytoHubba algorithms (MCC, Degree, MNC, EPC, and Closeness), followed by evaluation in the independent mouse ICH dataset GSE200575. Accordingly, the seven hubs identified here should be interpreted as context-dependent, transcriptomics-informed QR-associated candidate targets in acute experimental mouse ICH rather than as universal cross-species targets or experimentally confirmed direct molecular targets. Despite the limited gene-level overlap, the two studies may nevertheless converge functionally, because several prioritized genes in both studies are involved in inflammatory signaling, cellular stress responses, and secondary tissue injury.

qRT-PCR analysis of perihematomal tissue indicated that QR modulated hub-gene mRNA expression in a manner broadly consistent with the computational predictions. Following ICH induction, *Il1b* and *Mmp9* were markedly upregulated, consistent with activation of inflammatory and matrix-degrading cascades. QR treatment dose-dependently suppressed these increases, with the medium and high doses producing the most robust attenuation. This pattern is compatible with modulation of TNF- and IL-17-related inflammatory signaling, both of which are implicated in secondary injury after ICH (Zhang et al., 2021). Concurrently, QR increased *Timp1* expression, which may indicate enhanced ECM stabilization or repair-associated feedback. QR also elevated *Hmox1* at the high dose, suggesting engagement of a heme/oxidative-stress response after ICH (Chen et al., 2025; Chen-Roetling et al., 2015). However, the role of HO-1 in hemorrhagic stroke is context-dependent: although it may support heme catabolism and antioxidant defense, sustained or excessive HO-1 activity may also contribute to iron-mediated oxidative injury depending on cell type, timing, and downstream iron-handling capacity. Therefore, the *Hmox1* response observed here should be interpreted as a stress-response signature requiring further time-course and cell-specific validation, rather than as uniformly protective evidence. The transcriptional response also showed selectivity: *Fos* declined at the high dose, whereas *Myc* remained largely unchanged. This discrepancy suggests that *Myc* may represent a disease-associated transcriptomic hub rather than an acutely QR-responsive effector under the present experimental conditions. *Socs3* increased in a dose-dependent manner and peaked in the high-dose QR and DEX groups, which may reflect strengthened negative regulation of inflammatory signaling through the cytokine–JAK–STAT pathway (Ji et al., 2020).

In addition to brain transcriptional changes, systemic inflammation was evident after ICH. ELISA measurements showed increased serum IL-1β, TNF-α, IL-6, and MMP-9 in ICH mice, consistent with an enhanced peripheral inflammatory response that may exacerbate secondary brain injury after ICH. QR attenuated these elevations in a dose-dependent manner: the low dose had minimal impact, whereas the medium and high doses reduced all four mediators, with high-dose QR showing an anti-inflammatory profile comparable to DEX. DEX was used here as an anti-inflammatory comparator rather than as a clinically established disease-modifying therapy for ICH. Together, these data indicate that QR dampens the systemic inflammatory response after ICH, consistent with coordinated modulation of inflammatory and matrix-remodeling markers.

To examine whether the mouse-prioritized candidates were also relevant to human ICH pathophysiology, we analyzed paired perihematomal and remote brain tissues from patients with ICH, together with serum samples from patients with ICH and healthy controls. Human perihematomal tissues showed disease-relevant upregulation of *IL1B*, *MMP9*, *TIMP1*, and *HMOX1*, supporting the relevance of the prioritized hub genes in human ICH. The prominent *MMP9* upregulation is consistent with current evidence implicating MMP-9 in BBB disruption, hematoma progression, and perihematomal edema after ICH (Guan et al., 2025; Lattanzi et al., 2020). The concurrent upregulation of *TIMP1* may reflect a compensatory response to limit excessive proteolysis, whereas increased *HMOX1* mRNA is compatible with activation of a heme- and oxidative-stress-related response to hemoglobin breakdown after ICH (Chen et al., 2025; Chen-Roetling et al., 2015). The significant but comparatively moderate increase in *IL1B* is also consistent with the central role of IL-1-driven neuroinflammation in secondary brain injury after ICH (Taskin and Yao, 2025). These findings provide preliminary support for the disease relevance of several mouse-prioritized candidates in human ICH. However, because these patients were not treated with QR, the human tissue data do not establish QR-specific target engagement or therapeutic efficacy. Clinical translation will therefore require further evaluation of cross-species pharmacology, human pharmacokinetics and brain exposure, clinically achievable dosing, direct target engagement, therapeutic window, and safety.

Western blotting analysis further showed increased expression of IL-1β, MMP-9, and TIMP-1 in perihematomal tissues relative to matched remote tissues, consistent with the qRT-PCR findings. IHC provided additional spatial support for MMP-9 upregulation, with markedly stronger perihematomal immunoreactivity and a significantly larger MMP-9-positive area than in matched remote tissues. Together, these transcriptomic, proteomic, and histological findings support a prominent role for MMP-9-driven matrix remodeling in human perihematomal ICH tissue (Lattanzi et al., 2020; Guan et al., 2025).

At the systemic level, serum concentrations of all five cytokines—IL-1β, IL-6, IL-10, IL-17A, and TNF-α—were significantly elevated in ICH patients relative to healthy controls. The concurrent increases in IL-1β, TNF-α, and IL-10 suggest simultaneous pro- and anti-inflammatory activation, consistent with the biphasic peripheral immune response described after ICH (Dziedzic et al., 2002; Ohashi et al., 2023). The elevation of IL-10, a canonical anti-inflammatory cytokine, may reflect a compensatory regulatory response aimed at limiting systemic inflammatory injury, consistent with emerging evidence that IL-10 contributes to protection against BBB disruption and perihematomal edema after ICH (Xu et al., 2024). The increases in IL-6 and IL-17A further implicate cytokine-mediated amplification loops during the acute phase of human ICH, consistent with evidence for IL-6-mediated systemic inflammation (Dziedzic et al., 2002) and emerging evidence for IL-17-associated inflammatory activity in this setting (Taskin and Yao, 2025).

It is important to clarify the clinical context of the present findings. To date, no reliable clinical evidence supports the use of QR as a routine therapy for ICH, and QR is not included among the interventions recommended in current ICH management guidelines. Guideline-based management of spontaneous ICH primarily involves stroke unit or neurocritical care, acute blood-pressure management, rapid reversal of anticoagulation when indicated, management of complications, rehabilitation, and surgical intervention in appropriately selected patients (Greenberg et al., 2022). Therefore, the present results should be interpreted as preclinical evidence that QR may modulate inflammatory responses associated with secondary injury after ICH, rather than as support for QR as a replacement for current standard care or as a clinical recommendation.

Although several mouse-prioritized targets (*IL1B*, *MMP9*, *TIMP1*, and *HMOX1*) showed consistent aberrant expression in human perihematomal tissues, the patients did not receive QR treatment. These human tissue findings therefore support the disease relevance of the prioritized targets in human ICH pathology but do not establish target engagement or therapeutic efficacy of QR in humans. Translational application of QR will require further investigation of bioavailability, human pharmacokinetics, brain tissue exposure, route of administration and dose conversion, therapeutic time window, safety, and long-term functional outcomes. Until these issues are addressed, the present findings should be considered mechanistic and hypothesis-generating rather than clinically conclusive.

Several limitations should be noted. Database-dependent predictions may not capture species-, tissue-, cell-type-, sex-, or time-specific interactions, and the prioritized targets should therefore not be assumed to translate directly across species. The transcriptomic datasets were small and limited to acute-stage ICH. *In vivo* validation was restricted to male mice, a single collagenase-induced ICH model, and a single 24-h endpoint. The relatively small sample size (n = 6 per group) further limits the precision of effect estimates and the robustness and generalizability of the experimental findings. In the present animal experiments, brain water content, perihematomal gene expression, and serum inflammatory markers were evaluated at 24 h after ICH, without measurement of hematoma or lesion volume or neurological/behavioral outcomes. Therefore, the current results only support an association of QR with acute inflammatory, ECM-remodeling, and edema-related changes and do not allow conclusions regarding its effects on hematoma expansion, structural brain injury, sustained neuroprotection, or functional recovery. Additionally, the wet weight or volume of the dissected perihematomal tissue was not recorded during the mouse experiments, as the samples were immediately processed for RNA extraction; therefore, we cannot exclude potential variability arising from non-standardized tissue sampling. This limitation should be considered when interpreting the qRT-PCR results. Histopathological injury, BBB integrity, microglial activation, and direct oxidative-stress markers were not assessed in the mouse model, limiting mechanistic depth. The human cohort was small and observational, and the human samples support disease relevance rather than clinical validation of QR efficacy. Finally, docking and immune deconvolution analyses remain hypothesis-generating and require biochemical, cellular, and functional validation. Future studies incorporating multiple time points, quantitative hematoma/lesion assessment, long-term neurological and behavioral evaluations, histological and cell-specific analyses, and larger, appropriately designed cohorts are needed to further determine the therapeutic potential of QR in ICH.

## 5 Conclusion

QR was associated with attenuation of acute inflammatory and ECM-remodeling responses after experimental ICH, accompanied by changes in selected stress-response genes. Mouse experiments and human disease-context assessment support the disease relevance of these molecular signatures. Future studies should clarify direct target engagement, cell-specific mechanisms, oxidative-stress pathways, and long-term functional outcomes to better define the therapeutic potential of QR in ICH.

## Data Availability

The public transcriptomic datasets analyzed in this study are available in the NCBI Gene Expression Omnibus (GEO) under accession numbers GSE113151 and GSE200575.
