# Supplementary material for: Quercetin attenuates acute inflammation and extracellular matrix remodeling after intracerebral hemorrhage: transcriptomics-informed network pharmacology and experimental validation
Source: Front Pharmacol. 2026 Oct 19;17:1930434. doi: 10.3389/fphar.2026.1930434 (PMC13724369; doi:10.3389/fphar.2026.1930434)
Supplement: Supplementary file 1 [file Table10.docx]

Table S10 Results of GSEA for GSE113151

| ID | Set Size | Enrichment Score | NES | pvalue | p.adjust | qvalue |
| --- | --- | --- | --- | --- | --- | --- |
| ZHENG_IL22_SIGNALING_UP | 59 | 0.82085424 | 2.503173621 | 1.00E-10 | 7.15E-09 | 5.39E-09 |
| GROSS_HYPOXIA_VIA_ELK3_DN | 147 | 0.719419578 | 2.47968733 | 1.00E-10 | 7.15E-09 | 5.39E-09 |
| PLASARI_TGFB1_TARGETS_10HR_UP | 189 | 0.631089778 | 2.249134776 | 1.00E-10 | 7.15E-09 | 5.39E-09 |
| BIOCARTA_IL2RB_PATHWAY | 36 | 0.722764889 | 2.040314975 | 9.26E-05 | 1.57E-03 | 1.18E-03 |
| BROWN_MYELOID_CELL_DEVELOPMENT_UP | 167 | 0.758464026 | 2.656802902 | 1.00E-10 | 7.15E-09 | 5.39E-09 |
| MA_RAT_AGING_UP | 100 | 0.803137566 | 2.640753531 | 1.00E-10 | 7.15E-09 | 5.39E-09 |
| HESS_TARGETS_OF_HOXA9_AND_MEIS1_DN | 83 | 0.823367267 | 2.637528378 | 1.00E-10 | 7.15E-09 | 5.39E-09 |
| GALINDO_IMMUNE_RESPONSE_TO_ENTEROTOXIN | 78 | 0.836500836 | 2.6312953 | 1.00E-10 | 7.15E-09 | 5.39E-09 |
| ICHIBA_GRAFT_VERSUS_HOST_DISEASE_35D_UP | 148 | 0.746945003 | 2.579696448 | 1.00E-10 | 7.15E-09 | 5.39E-09 |
| WP_LUNG_FIBROSIS | 53 | 0.860573299 | 2.571491683 | 1.00E-10 | 7.15E-09 | 5.39E-09 |
| LIAN_LIPA_TARGETS_6M | 82 | 0.800450295 | 2.562991034 | 1.00E-10 | 7.15E-09 | 5.39E-09 |
| ZENG_GU_ICB_CONTROL_METAGENE_25_PRECICTIVE_ICB_RESPONSE | 192 | 0.707670725 | 2.52596184 | 1.00E-10 | 7.15E-09 | 5.39E-09 |
| SEKI_INFLAMMATORY_RESPONSE_LPS_UP | 77 | 0.801198989 | 2.51388003 | 1.00E-10 | 7.15E-09 | 5.39E-09 |
| SAUL_SEN_MAYO | 111 | 0.749352097 | 2.500297825 | 1.00E-10 | 7.15E-09 | 5.39E-09 |
| STEARMAN_TUMOR_FIELD_EFFECT_UP | 36 | 0.881357843 | 2.488011847 | 1.00E-10 | 7.15E-09 | 5.39E-09 |
| LIAN_LIPA_TARGETS_3M | 66 | 0.807406866 | 2.484416256 | 1.00E-10 | 7.15E-09 | 5.39E-09 |
| NEMETH_INFLAMMATORY_RESPONSE_LPS_UP | 89 | 0.747836118 | 2.412363462 | 1.00E-10 | 7.15E-09 | 5.39E-09 |
| LI_INDUCED_T_TO_NATURAL_KILLER_UP | 304 | 0.644278332 | 2.406458876 | 1.00E-10 | 7.15E-09 | 5.39E-09 |
| MCDOWELL_ACUTE_LUNG_INJURY_UP | 44 | 0.829566177 | 2.397025531 | 3.65E-10 | 2.24E-08 | 1.69E-08 |
| STEARMAN_LUNG_CANCER_EARLY_VS_LATE_DN | 59 | 0.782813816 | 2.387170341 | 3.17E-10 | 2.00E-08 | 1.51E-08 |

GSEA, Gene Set Enrichment Analysis.
