# Supplementary material for: Quercetin attenuates acute inflammation and extracellular matrix remodeling after intracerebral hemorrhage: transcriptomics-informed network pharmacology and experimental validation
Source: Front Pharmacol. 2026 Oct 19;17:1930434. doi: 10.3389/fphar.2026.1930434 (PMC13724369; doi:10.3389/fphar.2026.1930434)
Supplement: Supplementary file 2 [file Table11.docx]

Table S11 Results of GSVA for GSE113151

| ID |  | logFC | AveExpr | t | P.Value | adj.P.Val | B |
| --- | --- | --- | --- | --- | --- | --- | --- |
| BIOCARTA_NEUTROPHIL_PATHWAY |  | 1.30E+00 | -1.64E-02 | 9.39E+00 | 4.15E-08 | 7.27E-05 | 8.91E+00 |
| BIOCARTA_MSP_PATHWAY |  | 1.27E+00 | -1.85E-03 | 8.56E+00 | 1.53E-07 | 1.34E-04 | 7.68E+00 |
| BIOCARTA_MONOCYTE_PATHWAY |  | 1.20E+00 | 8.57E-04 | 8.25E+00 | 2.51E-07 | 1.46E-04 | 7.21E+00 |
| REACTOME_BH3_ONLY_PROTEINS_ASSOCIATE_WITH_AND_INACTIVATE_ANTI_APOPTOTIC_BCL_2_MEMBERS |  | 1.19E+00 | -1.06E-02 | 7.92E+00 | 4.40E-07 | 1.46E-04 | 6.68E+00 |
| REACTOME_TP53_REGULATES_TRANSCRIPTION_OF_GENES_INVOLVED_IN_G2_CELL_CYCLE_ARREST |  | 1.19E+00 | 1.04E-02 | 7.80E+00 | 5.42E-07 | 1.46E-04 | 6.48E+00 |
| BIOCARTA_LYMPHOCYTE_PATHWAY |  | 1.19E+00 | 8.34E-03 | 8.02E+00 | 3.72E-07 | 1.46E-04 | 6.84E+00 |
| REACTOME_TRIF_MEDIATED_PROGRAMMED_CELL_DEATH |  | 1.14E+00 | -7.28E-03 | 7.42E+00 | 1.04E-06 | 2.03E-04 | 5.85E+00 |
| REACTOME_G2_PHASE |  | 1.13E+00 | -2.05E-02 | 7.68E+00 | 6.68E-07 | 1.46E-04 | 6.28E+00 |
| BIOCARTA_FREE_PATHWAY |  | 1.08E+00 | -5.08E-02 | 7.28E+00 | 1.34E-06 | 2.35E-04 | 5.60E+00 |
| BIOCARTA_IFNG_PATHWAY |  | 1.04E+00 | 9.35E-03 | 6.64E+00 | 4.30E-06 | 5.21E-04 | 4.48E+00 |
| REACTOME_PROPIONYL_COA_CATABOLISM |  | -8.58E-01 | 6.50E-03 | -3.45E+00 | 3.06E-03 | 2.88E-02 | -1.92E+00 |
| REACTOME_THE_RETINOID_CYCLE_IN_CONES_DAYLIGHT_VISION |  | -8.64E-01 | 1.10E-02 | -4.34E+00 | 4.49E-04 | 8.40E-03 | -5.63E-02 |
| REACTOME_REGULATION_OF_CDH11_FUNCTION |  | -8.74E-01 | -1.38E-02 | -5.92E+00 | 1.74E-05 | 1.04E-03 | 3.12E+00 |
| REACTOME_UTILIZATION_OF_KETONE_BODIES |  | -8.91E-01 | -1.30E-01 | -3.72E+00 | 1.73E-03 | 2.17E-02 | -1.37E+00 |
| REACTOME_AMINO_ACID_CONJUGATION |  | -9.87E-01 | 1.71E-02 | -3.07E+00 | 6.95E-03 | 4.56E-02 | -2.70E+00 |
| REACTOME_CONJUGATION_OF_SALICYLATE_WITH_GLYCINE |  | -9.87E-01 | 1.71E-02 | -3.07E+00 | 6.95E-03 | 4.56E-02 | -2.70E+00 |
| REACTOME_WAX_AND_PLASMALOGEN_BIOSYNTHESIS |  | -9.90E-01 | -9.64E-03 | -6.68E+00 | 4.01E-06 | 5.21E-04 | 4.55E+00 |
| BIOCARTA_TUBBY_PATHWAY |  | -1.01E+00 | -7.82E-02 | -5.04E+00 | 1.03E-04 | 3.12E-03 | 1.38E+00 |
| REACTOME_SODIUM_COUPLED_SULPHATE_DI_AND_TRI_CARBOXYLATE_TRANSPORTERS |  | -1.03E+00 | 1.02E-01 | -4.12E+00 | 7.24E-04 | 1.17E-02 | -5.22E-01 |
| REACTOME_ATTENUATION_PHASE |  | -1.12E+00 | -1.43E-02 | -7.74E+00 | 6.04E-07 | 1.46E-04 | 6.37E+00 |

GSVA, Gene Set Variation Analysis.
