# Supplementary material for: Quercetin attenuates acute inflammation and extracellular matrix remodeling after intracerebral hemorrhage: transcriptomics-informed network pharmacology and experimental validation
Source: Front Pharmacol. 2026 Oct 19;17:1930434. doi: 10.3389/fphar.2026.1930434 (PMC13724369; doi:10.3389/fphar.2026.1930434)
Supplement: Supplementary file 3 [file Table12.docx]

Table S12 Molecular docking summary of quercetin (QR) with hub proteins.

| **Target**  **(gene/protein)** | **UniProt ID** | **Vina score**  **(kcal/mol)** | **Category** | **Key predicted H-bond residues**  **(receptor)** |
| --- | --- | --- | --- | --- |
| Fos | P01101 | -7.85 | Strong | GLU-304, ASP-302, ARG-144, ARG-140 |
| Mmp9 | P41245 | -7.46 | Strong | TYR-420, ALA-417, GLU-402, VAL-398 |
| Hmox1 | P14901 | -7.81 | Strong | ARG-136 |
| Il1b | P10749 | -7.57 | Strong | LYS-172, SER-169, PHE-163, GLU-99 |
| Myc | P01108 | -5.43 | Moderate | GLU-431 |
| Timp1 | P12032 | -7.27 | Strong | ARG-114, ASN-117, HIE-119, TYR-59, ASN-54, GLU-52 |
| Socs3 | O35718 | -6.69 | Moderate | TYR-166, ARG-109, TYR-165, LYS-91, GLN-105 |

Vina scores are reported in kcal/mol. Affinity categories were defined as weak (> -5), moderate (-7 to -5), or strong (< -7). Key hydrogen-bonding residues refer to receptor residues predicted to form hydrogen bonds with QR in the top-ranked pose.
