# Supplementary material for: Quercetin attenuates acute inflammation and extracellular matrix remodeling after intracerebral hemorrhage: transcriptomics-informed network pharmacology and experimental validation
Source: Front Pharmacol. 2026 Oct 19;17:1930434. doi: 10.3389/fphar.2026.1930434 (PMC13724369; doi:10.3389/fphar.2026.1930434)
Supplement: Supplementary file 4 [file Table13.docx]

Table S13. Lognormal Welch t-test with Holm–Šídák correction (n=6/group)

| Cytokine | HC GM | ICH GM | GMR (ICH/HC)  (95% CI) | Holm–Šídák p_adj |
| --- | --- | --- | --- | --- |
| IL-1β | 0.616 | 5.101 | 8.28 (4.76–14.41) | 0.000193 |
| IL-6 | 5.113 | 30.69 | 6.00 (1.97–18.26) | 0.014038 |
| IL-10 | 1.415 | 3.990 | 2.82 (2.03–3.91) | 0.000372 |
| IL-17A | 1.312 | 1.881 | 1.43 (1.12–1.83) | 0.014038 |
| TNF-α | 1.209 | 2.969 | 2.46 (1.89–3.19) | 0.000119 |
