# Supplementary material for: Quercetin attenuates acute inflammation and extracellular matrix remodeling after intracerebral hemorrhage: transcriptomics-informed network pharmacology and experimental validation
Source: Front Pharmacol. 2026 Oct 19;17:1930434. doi: 10.3389/fphar.2026.1930434 (PMC13724369; doi:10.3389/fphar.2026.1930434)
Supplement: Supplementary file 5 [file Table14.docx]

Supplementary Figure 1. Uncropped western blot images corresponding to Figure 14. The original blots show IL-1β, MMP-9, TIMP1, and β-actin in six paired remote and perihematomal human ICH tissue samples.

(A) IL-1β:

Exp 1	Exp 2	Exp 3

Exp 4	Exp 5	Exp 6

(B) MMP-9:

Exp 1	Exp 2	Exp 3

Exp 4	Exp 5	Exp 6

(C) TIMP1:

Exp 1	Exp 2	Exp 3

Exp 4	Exp 5	Exp 6

(D) β-actin:

Exp 1	Exp 2	Exp 3

Exp 4	Exp 5	Exp 6
