# Supplementary material for: Quercetin attenuates acute inflammation and extracellular matrix remodeling after intracerebral hemorrhage: transcriptomics-informed network pharmacology and experimental validation
Source: Front Pharmacol. 2026 Oct 19;17:1930434. doi: 10.3389/fphar.2026.1930434 (PMC13724369; doi:10.3389/fphar.2026.1930434)
Supplement: Supplementary file 6 [file Table4.docx]

Table S4 GEO Microarray Chip Information

|  | GSE113151 | GSE200575 |
| --- | --- | --- |
| Platform | GPL17021 | GPL24247 |
| Species | Mus musculus | Mus musculus |
| Tissue | Brain | Brain |
| Samples in CH group | 5 | 5 |
| Samples in Control group | 5 | 5 |
| Reference | / | PMID: 35677585 |

GEO, Gene Expression Omnibus; CH, Cerebral Hemorrhage.
