# Supplementary material for: Quercetin attenuates acute inflammation and extracellular matrix remodeling after intracerebral hemorrhage: transcriptomics-informed network pharmacology and experimental validation
Source: Front Pharmacol. 2026 Oct 19;17:1930434. doi: 10.3389/fphar.2026.1930434 (PMC13724369; doi:10.3389/fphar.2026.1930434)
Supplement: Supplementary file 7 [file Table6.docx]

Table S6 qPCR primers

| Gene | Forward Primer (5’-3) | Reverse Primer (5’-3’) |
| --- | --- | --- |
| Il1b  Mmp9 | GAAATGCCACCTTTTGACAGTG  GGACCCGAAGCGGACATTG | TGGATGCTCTCATCAGGACAG  CGTCGTCGAAATGGGCATCT |
| Timp1 | CTTGGTTCCCTGGCGTACTC | ACCTGATCCGTCCACAAACAG |
| Myc | TTCATCTGCGATCCTGACGAC | CACTGAGGGGTCAATGCACTC |
| Fos | CGGGTTTCAACGCCGACTA | TGGCACTAGAGACGGACAGAT |
| Hmox1 | GATAGAGCGCAACAAGCAGAA | CAGTGAGGCCCATACCAGAAG |
| Socs3 | TGCGCCTCAAGACCTTCAG | GCTCCAGTAGAATCCGCTCTC |
| β-Actin | GGCTGTATTCCCCTCCATCG | CCAGTTGGTAACAATGCCATGT |
