# Supplementary material for: Quercetin attenuates acute inflammation and extracellular matrix remodeling after intracerebral hemorrhage: transcriptomics-informed network pharmacology and experimental validation
Source: Front Pharmacol. 2026 Oct 19;17:1930434. doi: 10.3389/fphar.2026.1930434 (PMC13724369; doi:10.3389/fphar.2026.1930434)
Supplement: Supplementary file 8 [file Table7.docx]

Table S7 qPCR primers

| Gene | Forward Primer (5’-3) | Reverse Primer (5’-3’) |
| --- | --- | --- |
| IL-1β  MMP9 | ATGATGGCTTATTACAGTGGCAA  GGGACGCAGACATCGTCATC | GTCGGAGATTCGTAGCTGGA  TCGTCATCGTCGAAATGGGC |
| TIMP1 | AGAGTGTCTGCGGATACTTCC | CCAACAGTGTAGGTCTTGGTG |
| HMOX1 | AAGACTGCGTTCCTGCTCAAC | AAAGCCCTACAGCAACTGTCG |
| GAPDH | GGGAAACTGTGGCGTGAT | GAGTGGGTGTCGCTGTTGA |
