# Supplementary material for: Quercetin attenuates acute inflammation and extracellular matrix remodeling after intracerebral hemorrhage: transcriptomics-informed network pharmacology and experimental validation
Source: Front Pharmacol. 2026 Oct 19;17:1930434. doi: 10.3389/fphar.2026.1930434 (PMC13724369; doi:10.3389/fphar.2026.1930434)
Supplement: Supplementary file 9 [file Table9.docx]

Table S9 Results of GO and KEGG Enrichment Analysis for Key Genes

| ONTOLOGY | ID | Description | GeneRatio | BgRatio | pvalue | p.adjust | qvalue |
| --- | --- | --- | --- | --- | --- | --- | --- |
| BP | GO:0071621 | granulocyte chemotaxis | 7/28 | 135/28891 | 4.54E-11 | 4.80E-08 | 2.01E-08 |
| BP | GO:0030595 | leukocyte chemotaxis | 8/28 | 240/28891 | 5.43E-11 | 4.80E-08 | 2.01E-08 |
| BP | GO:0097529 | myeloid leukocyte migration | 8/28 | 253/28891 | 8.27E-11 | 4.87E-08 | 2.04E-08 |
| BP | GO:0097530 | granulocyte migration | 7/28 | 167/28891 | 2.03E-10 | 8.98E-08 | 3.75E-08 |
| BP | GO:0045766 | positive regulation of angiogenesis | 7/28 | 182/28891 | 3.71E-10 | 1.09E-07 | 4.57E-08 |
| CC | GO:0062023 | collagen-containing extracellular matrix | 5/28 | 396/28573 | 3.77E-05 | 3.32E-03 | 2.66E-03 |
| CC | GO:0030141 | secretory granule | 5/28 | 498/28573 | 1.11E-04 | 4.89E-03 | 3.92E-03 |
| CC | GO:0031091 | platelet alpha granule | 2/28 | 21/28573 | 1.92E-04 | 5.64E-03 | 4.52E-03 |
| MF | GO:0005125 | cytokine activity | 5/27 | 236/28407 | 2.64E-06 | 3.48E-04 | 1.61E-04 |
| MF | GO:0001046 | core promoter sequence-specific DNA binding | 3/27 | 51/28407 | 1.55E-05 | 1.00E-03 | 4.64E-04 |
| MF | GO:0050840 | extracellular matrix binding | 3/27 | 58/28407 | 2.28E-05 | 1.00E-03 | 4.64E-04 |
| MF | GO:0000979 | RNA polymerase II core promoter sequence-specific DNA binding | 2/27 | 23/28407 | 2.17E-04 | 5.98E-03 | 2.76E-03 |
| MF | GO:0019865 | immunoglobulin binding | 2/27 | 26/28407 | 2.79E-04 | 5.98E-03 | 2.76E-03 |
| KEGG | mmu04668 | TNF signaling pathway - Mus musculus (house mouse) | 7/24 | 118/10526 | 5.50E-09 | 7.75E-07 | 4.75E-07 |
| KEGG | mmu05418 | Fluid shear stress and atherosclerosis - Mus musculus (house mouse) | 6/24 | 149/10526 | 7.93E-07 | 5.59E-05 | 3.42E-05 |
| KEGG | mmu05323 | Rheumatoid arthritis - Mus musculus (house mouse) | 5/24 | 87/10526 | 1.29E-06 | 6.06E-05 | 3.71E-05 |
| KEGG | mmu04657 | IL-17 signaling pathway - Mus musculus (house mouse) | 5/24 | 94/10526 | 1.90E-06 | 6.68E-05 | 4.09E-05 |
| KEGG | mmu05417 | Lipid and atherosclerosis - Mus musculus (house mouse) | 6/24 | 217/10526 | 7.07E-06 | 1.99E-04 | 1.22E-04 |

GO, Gene Ontology; BP, Biological Process; CC, Cellular Component; MF, Molecular Function; KEGG, Kyoto Encyclopedia of Genes and Genomes.
